# Inhibition of cancer stem cell like cells by a synthetic retinoid

**DOI:** 10.1038/s41467-018-03877-7

**Published:** 2018-04-11

**Authors:** Junwei Chen, Xin Cao, Quanlin An, Yao Zhang, Ke Li, Wenting Yao, Fuchun Shi, Yanfang Pan, Qiong Jia, Wenwen Zhou, Fang Yang, Fuxiang Wei, Ning Wang, Biao Yu

**Affiliations:** 1 Laboratory for Cellular Biomechanics and Regenerative Medicine, Department of Biomedical Engineering, College of Life Science and Technology, Huazhong University of Science and Technology, 1037 Luoyu Road, Wuhan, Hubei 430074 China; 2 State Key Laboratory of Bioorganic and Natural Products Chemistry, Center for Excellence in Molecular Synthesis, Shanghai Institute of Organic Chemistry, Chinese Academy of Sciences, 345 Lingling Road, Shanghai, 200032 China; 3 Department of Mechanical Science and Engineering, University of Illinois at Urbana-Champaign, Urbana, IL 61801 USA

## Abstract

Developing novel drugs that can abrogate the growth and metastasis of malignant tumors is a major challenge for cancer researchers. Here we describe a novel synthetic retinoid, namely WYC-209, which inhibits proliferation of malignant murine melanoma tumor-repopulating cells (TRCs), known to resist conventional drug treatment, with an IC_50_ of 0.19 μM in a dose-dependent manner. WYC-209 also inhibits proliferation of TRCs of human melanoma, lung cancer, ovarian cancer, and breast cancer in culture. Interestingly, the treated TRCs fail to resume growth even after the drug washout. Importantly, the molecule abrogates 87.5% of lung metastases of melanoma TRCs in immune-competent wild-type C57BL/6 mice at 0.22 mg kg^−1^ without showing apparent toxicity. Pretreating the melanoma TRCs with retinoic acid receptor (RAR) antagonists or with RAR siRNAs blocks or reduces the inhibitory effect of the molecule, suggesting that the target of the molecule is RAR. WYC-209 induces TRC apoptosis and pretreating the TRCs with caspase 3 inhibitor or depleting caspase 3 with siRNAs substantially rescues growth of TRCs from WYC-209 inhibition, suggesting that WYC-209 induces TRCs apoptosis primarily via the caspase 3 pathway. Our findings demonstrate the promise of the new retinoid WYC-209 in treating malignant melanoma tumors with high efficacy and little toxicity.

## Abstract (web summary)

The discovery of efficient drugs is a main challenge in cancer therapy. Here, the authors report a synthetic retinoid, WYC-209 with anti-tumor and anti-metastatic activity mediated by the retinoic acid receptor signaling pathway.

## Introduction

Chemotherapy is one of the principal modes of treatment for cancer, but resistance to chemotherapeutic drugs is a hallmark of malignant tumors that results in major limitation in chemotherapy^1,2^. Cancer stem cells (CSCs) or tumor-initiating cells (TICs) are a self-renewing, highly tumorigenic subpopulation of tumor cells. They play a critical role in the initiation and progression of cancer^3^. CSCs or TICs exhibit high chemo-resistance to conventional chemotherapeutic drug treatment and therefore are speculated to be the key players in cancer relapse after chemotherapy^4^. As a consequence, developing targeted chemotherapeutic drugs to abrogate CSCs or TICs is a key task in cancer research and clinical application. We have recently developed a mechanical method of selecting and growing tumorigenic cells from cancer cell lines and primary cancer cells by culturing single cancer cells in soft fibrin gels^5^. The selected cancer cells display high self-renewal ability and are resistant to chemotherapeutic drugs such as cisplatin and doxorubicin^5^. Remarkably, when injected the selected cancer cells into tail veins, as few as ten of such cells can generate distant metastatic colonization in immune-competent mice. We thus functionally define these soft-fibrin-gel-selected cancer cells as tumor-repopulating cells (TRCs), differentiating them from CSCs or TICs that are selected via cell surface stem cell marker approaches. These TRCs express high levels of self-renewing gene *Sox2* and low levels of master differentiation gene *Mitf* and hence appear to remain undifferentiated or partially differentiated^6^. Treating TRCs with retinoid acid (RA), which is a nonspecific differentiation factor, could inhibit TRCs extravasation^7^, a key late stage in metastasis. However, poor water solubility and high toxicity of RA significantly limit its use in clinical treatment of cancer^8–11^. In order to develop highly potent retinoids with great efficiency in inhibition of cancer stem cell like TRCs, we have performed in-house drug discovery processes to specifically overcome these limitations. In the current study, we describe synthesis and discovery of a novel retinoid, named WYC-209, which abrogates growth of TRCs of several cancer cell lines in culture and inhibits lung metastasis by melanoma TRCs in vivo, with little toxicity on non-cancerous cells or immune-competent mice.

## Results

### Retinoid library screening and discovery of WYC-209

**Fig. 1:**
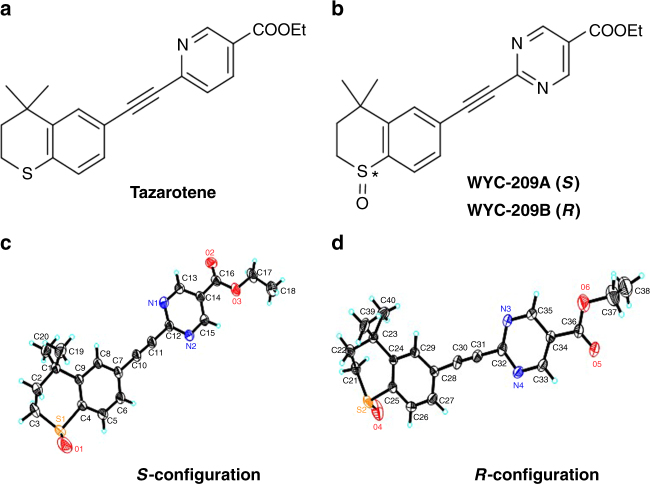
Structures of Tazarotene and WYC-209 and X-ray crystal structures of WYC-209A and WYC-209B. **a** Structure of Tazarotene. **b** Structures of WYC-209A (*S*) and WYC-209B (*R*). **c**, **d** X-ray crystal structures of WYC-209A and WYC-209B. The configurations of the sulfoxides were characterized by their crystal structures. Thermal ellipsoids were at 30% probability. Key bond lengths [Å] and angles [°]: S(1)-O(1) 1.422(7), S(1)-C(1) 1.768(8), S(1)-C(5) 1.791(6), S(2)-O(4) 1.447(6), S(2)-C(25) 1.802(7), S(2)-C(21) 1.831(10), O(1)-S(1)-C(1) 113.3(4), O(1)-S(1)-C(5) 108.4(4), C(1)-S(1)-C(5) 97.3(3), O(4)-S(2)-C(21) 99.2(5), O(4)-S(2)-C(25) 107.6(3), C(25)-S(2)-C(21) 96.5(4)

Retinoic acid and its analogs, referred to as retinoids, bind retinoic acid receptors that possess functional characteristics of a tumor suppressor^12^. According to the clarified SARs (Structure Activity Relationships) of synthetic retinoids, an aryl carboxylic acid right half which that mimics RA’s terminal acid group is the most important pharmacophore to retinoids^13^. However, since most retinoids are highly lipophilic compounds with propensity to accumulate in the human body, researchers have identified RA analogs that substitute benzoic acid with aromatic rings; for example, Tazarotene^14^ (Fig. 1a), Am80P^15^, and LG100268^16^. Following these rationales, we have established a synthetic retinoid library via a parallel synthesis manner. These synthetic retinoids were screened using the developed 3D B16-F1 TRCs colony model^5,6^. One of the compounds, namely WYC-209, was a racemic sulfoxide derivative bearing a 5-pyrimidine-acid skeleton (MW = 368.1, Fig. 1b; Supplementary Figs. 1–5; see Methods) with improved water solubility (Supplementary Table 1). Enantiomers WYC-209A (Fig. 1c) and WYC-209B (Fig. 1d) were prepared using Kagan’s asymmetric sulfide oxidation conditions with Ti (O*i*-Pr)_4_ and (+/−)-DET^17^ (Supplementary Figs. 1 and 6). WYC-209A acid binds human RARα, RARβ, and RARγ with *K*_*d*_ of 5.3, 2.5, and 0.53 nM, respectively; WYC-209B acid binds human RARα with *K*_*d*_ of 1.3 nM. These values are in the same order of magnitude as those *K*_*d*_ values of all-trans retinoic acid (ATRA) (Supplementary Table 2).

### Inhibition of TRC colony growth by WYC-209

**Fig. 2:**
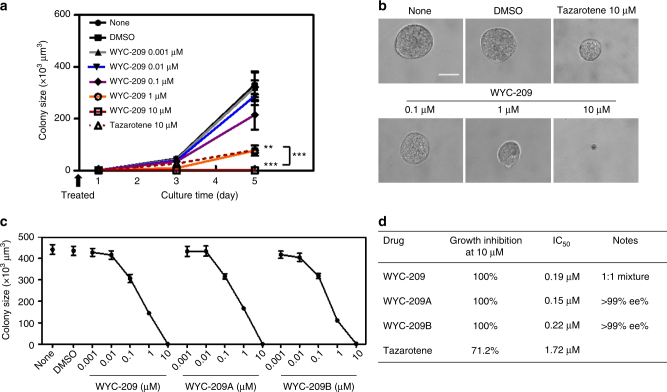
Colony growth of TRCs was abrogated by WYC-209. **a** None: non-treated B16-F1 cells cultured in 90-Pa fibrin gels. DMSO: Control B16-F1 cells cultured in 90-Pa fibrin gels and treated with 0.1% DMSO (dimethyl sulfoxide, solvent for WYC-209) on day 0. WYC-209 0.001 μM, WYC-209 0.01 μM, WYC-209 0.1 μM, WYC-209 1 μM and WYC-209 10 μM: Control B16-F1 cells were cultured in 90-Pa fibrin gels treated with WYC-209 on day 0 with various concentrations. Tazarotene 10 μM: Control B16-F1 cells were cultured in 90-Pa fibrin gels treated with 10 μM Tazarotene on day 0 as positive control. *P* < 0.002 between DMSO and WYC-209 1 µM; *P* < 0.0004 between DMSO and WYC-209 10 µM on day 5; *P* < 1.1e^−7^ between WYC-209 10 µM and Tazarotene 10 μM on day 5. Mean ± s.e.m.; ^**^P < 0.01; ^***^P < 0.001; at least three independent experiments. **b** Representative images of colonies on day 5 in **a**; Scale bar, 50 μm. **c** Dose-effect curves of WYC-209, WYC-209A, and WYC-209B. Quantified colony sizes on day 5 after B16-F1 cells were cultured in 90-Pa fibrin gels and treated with different conditions. None: medium without drugs. DMSO: medium with 0.1% DMSO. Mean ± s.e.m.; three separate experiments. **d** IC_50_ of WYC-209, WYC-209A, WYC-209B, and Tazarotene. Student’s *t*-test was used in all statistics

WYC-209, WYC-209A, and WYC-209B all demonstrated excellent growth inhibition activity for B16-F1 TRCs in a dose-dependent manner (Fig. 2a–c), with 100% inhibition at 10 μM (Fig. 2d). In contrast, at 10 μM, Tazarotene inhibited B16 growth by 71% and ATRA inhibited B16 growth by 80% (Supplementary Fig. 7), which is consistent with the proliferation data (Supplementary Fig. 8). The strong inhibitory activity of WYC-209 is unexpected because it is known that after oxidation of the sulfur atom on Tazarotene, the sulfoxide derivative AGN 190844 loses its activity^18^. In addition to its inhibitory effect on growth of B16-F1 TRCs, WYC-209 also completely inhibited the proliferation of unselected B16-F1 melanoma cells cultured on 2D rigid dishes (Supplementary Fig. 9); in contrast, this compound showed little impact toward the non-cancerous murine cell line 3T3 fibroblasts on rigid dishes (Supplementary Fig. 10), suggesting that this compound is not toxic for non-cancerous cells in culture. These dose-response curves indicated that both enantiomers at 10 µM abrogated TRC growth via inducing apoptosis of the TRCs (Supplementary Figs. 11 and 12), with IC_50_ of 0.15 and 0.22 μM on B16-F1 TRCs, respectively, which were about 10-fold lower than that of Tazarotene (Fig. 2d). Furthermore, cell cycle analyses show that WYC-209, WYC-209A and WYC-209B decreased S phase of TRCs in a dose-dependent manner, and at 10 μM they completely abolished S phase of TRCs, compared with 10 μM of Tazarotene and ATRA which could only decrease ~45% and 55% of the S phase of TRCs (Supplementary Fig. 13). Taken together, these data suggest that both WYC-209A and WYC-209B could abrogate proliferation of B16-F1 TRCs in culture and induce apoptosis with similar efficiency.

### Long-term effects of WYC-209

**Fig. 3:**
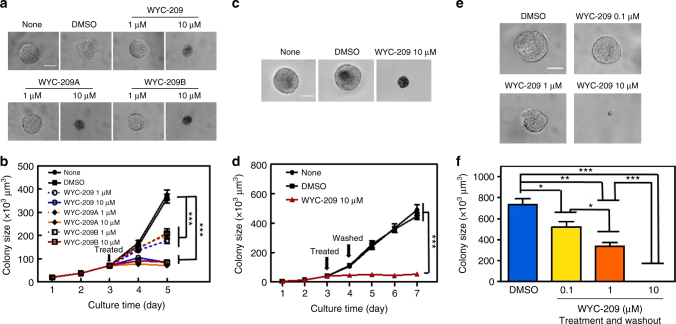
Inhibition of tumor cell colony growth by WYC-209 with or without washout. **a** Murine melanoma cells were cultured in 90-Pa fibrin gels for 3 days and then treated with medium alone (None), DMSO (0.1% DMSO-containing medium), 1 or 10 μM WYC-209, 1 or 10 μM WYC-209A, WYC-209B. Representative images of colonies on day 5; Scale bar, 50 μm. **b** Summarized data. Mean ± s.e.m.; *n* = 15 samples; three separate experiments; ^***^P < 0.001. **c** Representative images of colonies on day 7: None: untreated; DMSO: treated with 0.1% DMSO as a vehicle; WYC-209 10 μM: 3 days after WYC-209 was washed out; Scale bar, 50 μm. **d** Drugs were added on day 3 and washed out on day 4; the cells were cultured till day 7. Mean ± s.e.m.; *n* = 15 samples; three separate experiments; ^***^P < 0.001. **e** WYC-209 abrogates colony formation of TRCs. DMSO: Control B16-F1 cells, cultured in 90-Pa 3D fibrin gels and treated with 0.1% DMSO on day 0 for 5 days, were then re-plated into 90-Pa 3D fibrin gels as single individual cells for 5 days without treatment. WYC-209 0.1 μM, 1 μM, or 10 μM: Control B16-F1 cells, cultured in 90-Pa 3D fibrin gels and pretreated with WYC-209 on day 0 with various concentrations for 5 days, then after washing out WYC-209, the cells were re-plated into 90-Pa 3D fibrin gels for 5 days. Representative images of colonies on day 5 (all colonies started as a single cell on day 0); Scale bar, 50 μm. **f** Summarized colony sizes on day 5. Mean ± s.e.m.; *n* = 15 samples; three separate experiments; ^*^P < 0.05; ^**^P < 0.01; ^***^P < 0.001. Student’s *t*-test was used in all statistics

To explore whether WYC-209 could inhibit growth of TRCs after colonies already formed, we cultured B16-F1 cells into 3D soft fibrin gels for 3 days and then treated the cells with WYC-209 on day 3. As shown in Fig. 3a, b, WYC-209A, WYC-209B, and WYC-209 inhibited the growth of TRCs in a dose-dependent manner (Fig. 3a, b). To explore whether the effect of WYC-209 has any long-term effects, we cultured B16-F1 cells into 90 Pa 3D soft fibrin gel for 3 days, treated with WYC-209, WYC-209A, or WYC-209B, and then washed out the compound on day 4. TRCs failed to resume growing even 3 days after 10 µM compound washout (Fig. 3c, d; Supplementary Fig. 14). Importantly, after 10 µM WYC-209, WYC-209A, or WYC-209B treated melanoma TRCs were re-plated back into new soft fibrin gels after washing out the compounds in the medium, without any soluble WYC-209, WYC-209A, or WYC-209B in the medium, none of the cells grew at all for at least 5 days compared with the control group; the TRC growth-blocking effects of WYC-209, WYC-209A, or WYC-209B were dose-dependent, as there was some tumor cell growth when the cells were pretreated with 0.1 or 1 μM of the compound that was then washed out (Fig. 3e, f; Supplementary Fig. 15). These results are consistent with the data that 0.1 and 1 μM compounds did not induce TRC apoptosis (Supplementary Fig. 12) and inhibited cell proliferation via blocking S phase of the cell cycle (Supplementary Fig. 13). Together, our data suggest that the effects of WYC-209, WYC-209A, or WYC-209B are long lasting and there is no sign of “relapse” in this in vitro cell culture model.

### WYC-209 abrogates growth of human TRCs

**Fig. 4:**
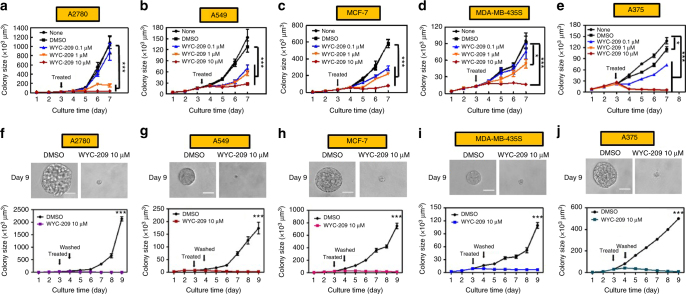
WYC-209 inhibits colony growth of five different human cancer lines. Human ovarian cancer A2780 (**a**), human lung cancer A549 (**b**), human breast cancer MCF-7 (**c**), human melanoma MDA-MB-435s (**d**), and human malignant melanoma A375 (**e**) were cultured in 90-Pa fibrin gels for 3 days and treated with 0.1, 1, or 10 μM WYC-209. **f**–**j** A2780, A549, MCF-7, MDA-MB-435s, and A375 were cultured in 90-Pa fibrin gels for 3 days, treated with 10 μM WYC-209, and were then washed out of the compound on day 4. None: cell medium only, DMSO: medium with 0.1% DMSO. Note that because the cell doubling time was different for different types of cancer cells, the colony sizes varied. Mean ± s.e.m.; *n* = 15 samples; three separate experiments; ^*^P < 0.05; ^**^P < 0.01; ^***^P < 0.001. Student’s *t*-test was used in all statistics

To determine whether WYC-209 inhibitory effect could be generalized to other types of TRCs, we examined effects of WYC-209 treatment in five different human cancer cell lines, including human ovarian carcinoma A2780, human lung adenocarcinoma A549, human breast cancer MCF-7, human melanoma MDA-MB-435s, and human malignant melanoma A375. WYC-209 inhibited all five types of TRCs in a dose-dependent manner when the compound was added on day 3 (Fig. 4a–e; Supplementary Fig. 16). Treating these TRCs on day 0 (i.e., the compound was added at the same time when the cells were seeded into the soft fibrin gels) also blocked their growth in a dose-dependent manner, with IC_50_ ranging from 0.10 to 0.52 μM, respectively (Supplementary Table 3). WYC-209, WYC-209A, or WYC-209B showed better inhibitory effect than Tazarotene and ATRA on cell growth and proliferation (Supplementary Figs. 7 and 8). All human TRCs growths were 100% inhibited at 10 μM WYC-209 (Supplementary Fig. 17). In addition, when human cancer cells were treated with WYC-209 on day 3 and the compound was washed out on day 4, TRCs failed to resume growth 5 days after the drug washout (Fig. 4f–j; Supplementary Fig. 18). Furthermore, when the pretreated human TRCs were isolated and re-plated back into the soft fibrin gels after washing out the compounds in the medium, even without any soluble WYC-209, WYC-209A, or WYC-209B in the medium, the colony growth was inhibited in a dose-dependent manner: partially inhibited at 0.1 or 1 μM and completely inhibited at 10 μM (Supplementary Fig. 19). WYC-209, WYC-209A, or WYC-209B also inhibited growth of unselected human cancer cells on 2D rigid dishes in a dose-dependent manner and completely blocked cell growth at 10 μM (Supplementary Fig. 20). In contrast, these compounds showed much less inhibitory effects towards the non-cancerous human epidermal HaCaT cells on 2D rigid dishes and in 3D soft fibrin gels (Supplementary Fig. 21). Together, our data suggest that WYC-209 is able to inhibit and block growth of TRCs of both murine and human tumor cells in culture with a long-lasting effect.

### WYC-209 inhibits tumor metastasis in vivo

**Fig. 5:**
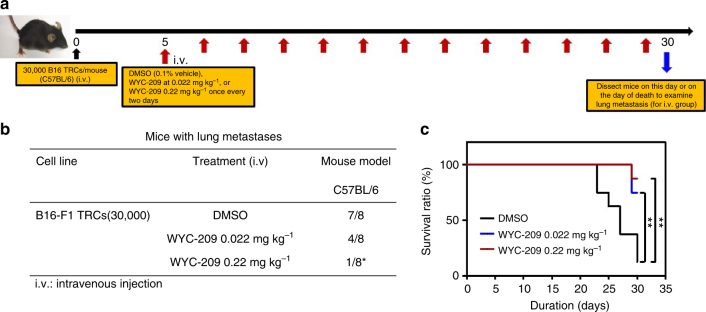
WYC-209 inhibits tumor metastasis in vivo. **a** Schematic of the experimental protocol; i.v., intravenous injection. Control B16-F1 cells were cultured in 90-Pa fibrin gels for 5 days to form TRCs. TRCs were isolated from gels and injected intravenously via tail veins into wild-type immune-competent syngeneic C57BL/6 mice at 30,000 cells per mouse. Three groups of mice were treated in this experiment, 8 mice each group. DMSO group: 8 mice were injected with TRCs for 5 days and then were treated with DMSO (0.1%, vehicle) every 2 days via intravenous injection. WYC-209 0.022 mg kg^−1^: 8 mice were injected with TRCs for 5 days then treated with WYC-209 0.022 mg kg^−1^ every 2 days by intravenous injection. WYC-209 0.22 mg kg^−1^: 8 mice were injected with TRCs for 5 days and then were treated with WYC-209 0.22 mg kg^−1^ every 2 days by intravenous injection. Mice were dissected on day 30 or on the day of death to examine signs of lung metastases. **b** Lung metastasis frequency of B16-F1 TRCs after treated with WYC-209. ^*^P < 0.05 with Fisher’s exact test. **c** Survival ratio of C57BL/6 mice transplanted with TRCs treated with WYC-209 or DMSO in protocol (**a**). Statistical analyses of survival ratio in **c** were performed by Mantel–Cox test (^**^P < 0.01 compared to DMSO-treated group)

To determine whether WYC-209 is effective in inhibition of tumor metastasis^19,20^ in vivo, we injected intravenously via tail veins 30,000 B16-F1 TRCs into female immune-competent C57BL/6 mice to form lung metastases. To simulate early cancer metastasis in human patients, we chose a 5-day waiting period after injection of melanoma TRCs to allow for plenty of time for the malignant TRCs to form micrometastases and to grow into metastatic colonies in the lungs. Five days later, 0.022 or 0.22 mg kg^−1^ WYC-209 were intravenously injected into mice once every two days for 25 days. 0.1% DMSO was injected as a negative control. On day 30, all three groups of mice were sacrificed for tumor examination since most mice from the DMSO-treated group died (Fig. 5). Seven out of eight mice treated with DMSO formed lung metastases; four out of eight mice formed lung metastases when the mice were treated with 0.022 mg kg^−1^ WYC-209; only one out of eight mice formed lung metastases when 0.22 mg kg^−1^ WYC-209 were treated (Fig. 5, Supplementary Fig. 22). After treating with 0.022 or 0.22 mg kg^−1^ WYC-209, the average mouse weight was similar to that in the control group (Supplementary Fig. 23a), suggesting that the compound did not cause weight loss in the mice. Furthermore, the average lung weight was less in the 0.22 mg kg^−1^ WYC-209 treated group than that in the DMSO group (Supplementary Fig. 23b), consistent with the data that most lungs were tumor-free in this WYC-209 treated group. There were no observable toxic or necrotic effects or weight loss on organs of liver and stomach after WYC-209 treatment (Supplementary Figs. 24 and 25). Similar results were observed in male mice (Supplementary Fig. 26), suggesting that there is no gender difference in inhibition of TRC metastasis by the compound.

To explore whether the enantiomers of WYC-209 are effective in inhibition of tumor metastasis, we examined WYC-209A and WYC-209B using the same protocol as WYC-209. Both WYC-209A and WYC-209B showed effective inhibition of tumor metastasis: all eight mice treated with DMSO formed lung metastases; three out of eight mice formed lung metastases when the mice were treated with 0.022 mg kg^−1^ WYC-209A or WYC-209B; only one out of eight mice formed lung metastases when 0.22 mg kg^−1^ WYC-209A or WYC-209B were treated (Supplementary Fig. 27). Hematoxylin and eosin (H&E) staining of lung, liver, stomach, spleen, and bone confirmed the above results and no metastases were found in any organs except the lungs in the DMSO group and the 0.022 mg kg^−1^ compound group (Supplementary Fig. 28). To explore the effects of treating TRCs with WYC-209 in fibrin gels then injecting these cells into untreated mice, we seeded B16-F1 cells into 3D fibrin gel, treated with 10 µM WYC-209 or with 0.1% DMSO as control on day 3. On day 5, TRCs were isolated and injected intravenously via tail veins into immune-competent C57BL/6 mice at 30,000 TRCs per mouse without any further treatment with the compound. All eight mice formed lung metastases when they were injected with DMSO-pretreated TRCs. In contrast, there were no lung metastases in mice injected with WYC-209-pretreated TRCs (Supplementary Fig. 29). Taken together, these data demonstrate that WYC-209 is an effective drug in inhibiting lung metastases in wild-type mice with little toxicity.

### Mechanism of WYC-209 action

**Fig. 6:**
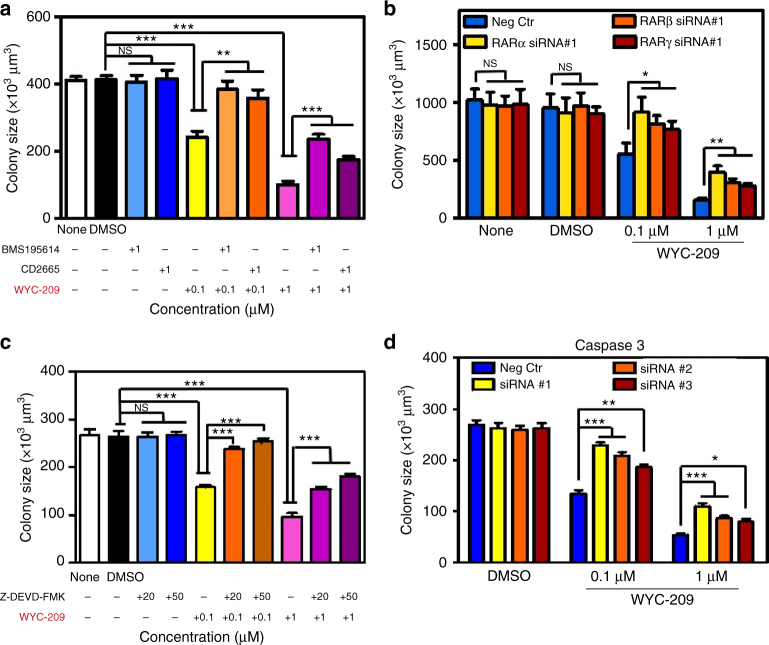
WYC-209 inhibits TRCs growth by binding RA receptors and via the caspase 3 pathway. **a** Control B16-F1 cells were cultured in 90-Pa fibrin gels treated with RA receptor antagonists for 3 h, then treated with 0.1 or 1 μM WYC-209, and cultured for 5 days. Summarized colony sizes after drug treatments on day 5 were compared with DMSO groups. None: medium without drugs. DMSO: medium with 0.1% DMSO. BMS 195614: Retinoic acid receptor (RAR) α-selective antagonist. CD 2665: selective RARβ/γ antagonist. **b** Control B16-F1 cells were transfected with negative control (scrambled siRNA), RARα, RARβ, or RARγ siRNA #1 s, respectively, and were then re-plated in soft fibrin gels, and were treated with 0.1 or 1 μM WYC-209. Summarized colony sizes after drug treatments on day 5 were compared with Negative control groups. None: medium without drugs. DMSO: medium with 0.1% DMSO. Neg Ctr: negative control. **c** Control B16-F1 cells were cultured in 90-Pa fibrin gels treated with caspase 3 inhibitor Z-DEVD-FMK for 3 h, then treated with 0.1 or 1 μM WYC-209, and cultured for 5 days. Summarized colony sizes after drug treatments on day 5 were compared with DMSO groups. None: medium without drugs. DMSO: medium with 0.1% DMSO. Z-DEVD-FMK: caspase 3 inhibitor. **d** Control B16-F1 cells were transfected with negative control (scrambled siRNA), caspase 3 siRNA #1, #2, or #3, respectively. The cells were then re-plated in soft fibrin gels and treated with 0.1 or 1 μM WYC-209. Summarized colony sizes after drug treatment on day 5 were compared with Negative control groups. DMSO: medium with 0.1% DMSO. Neg Ctr: negative control. Mean ± s.e.m.; *n* = 15 samples; three separate experiments; ^*^P < 0.05; ^**^P < 0.01; ^***^P < 0.001. Student’s *t*-test (plus Bonferroni correction when appropriate) was used for statistics

Retinoic acid receptor (RAR) is presumed to function as a tumor suppressor in various contexts since its absence is associated with tumorigenicity and its presence causes cell cycle arrest^21,22^. Tazarotene binds all three members of the RAR family. As WYC-209 has a similar skeleton to Tazarotene, we suspected that the ethyl ester of WYC-209 would be quickly hydrolyzed with esterases and its acid form could enter the nucleus and bind to RARs. To explore this possibility, we cultured B16-F1 into 3D soft fibrin gels and treated them with RAR antagonists for 3 h before adding WYC-209. Pre-treating with RARα antagonist BMS195614, or RARβ/γ antagonist CD2665 rescued the TRC growth from the inhibitory effects of WYC-209 in a dose-dependent manner (Fig. 6a) and the rescuing effect was minimal from the inhibitory effects of Tazarotene (Supplementary Fig. 30), while adding these antagonists alone had no impact on TRC growth. Pre-treating with RAR pan-antagonist AGN-194310 also rescued the TRC growth from the inhibitory effects of WYC-209, WYC-209A, or WYC-209B in a dose-dependent manner (Supplementary Fig. 31). Consistent with the data of antagonists treatments, depleting RARα, RARβ, or RARγ separately with specific siRNAs (Supplementary Figs. 32 and 33) also rescued the TRC growth from the inhibitory effects of WYC-209 (Fig. 6b), suggesting that RARs are a primary cellular target for WYC-209.

### WYC-209 induces TRC apoptosis via caspase 3 pathway

Since WYC-209 can abrogate TRC growth by inducing TRC apoptosis, we explored how WYC-209 induces TRC apoptosis. It is known that caspase 3 is an important player in apoptotic pathway^23,24^. We cultured B16-F1 or A375 into 3D soft fibrin gels and treated them with the caspase 3 inhibitor z-DEVD-FMK for 3 h before adding WYC-209. Pretreating with z-DEVD-FMK substantially rescued growth of mouse melanoma B16-F1 TRCs (Fig. 6c) and human melanoma A375 TRCs (Supplementary Fig. 34) from WYC-209 inhibition in a dose-dependent manner, but could not rescue growth of TRCs from the inhibitory effects of Tazarotene (Supplementary Fig. 35). Consistent with the result of the specific inhibitor, knocking down caspase 3 expression by siRNAs (Supplementary Fig. 36) also rescued the TRCs growth from the inhibitory effects of WYC-209 (Fig. 6d and Supplementary Fig. 34). Furthermore, 10 μM of WYC-209, WYC-209A, or WYC-209B led to an increase of cleaved (active) caspase-3 and PARP (Supplementary Fig. 37). Together these results suggest that WYC-209 induces TRC apoptosis primarily via the caspase 3 pathway.

### WYC-209 exhibits little systemic toxicity

For any novel anti-cancer compound to be useful in vivo, potential systemic toxicity should be examined. We evaluated the potential systemic toxicity of WYC-209 in order to determine druggability of WYC-209. The cardiac toxicity assays towards hERG were tested in the CHO hERG cell line using a conventional patch clamp system. The cells were transfected with hERG cDNA and showed stable hERG channel expression. Compared with the positive control drug cisapride (IC50 = 15 nM), both WYC-209A and WYC-209B showed negligible hERG channel blocking activity (IC_50_ > 30 μM) (Supplementary Table 4). These results suggest that both isomers of WYC-209 were cardiacally safe at high efficacious doses. We also evaluated the possibility of WYC-209 in inhibiting human liver microsomes. Five representative metabolic enzymes, including cytochromes P450 (CYP) 1A2, 2C9, 2C19, 2D6, and 3A4, were selected and tested with each isomer using well-established specific inhibiting systems. As shown in Supplementary Table 5, both WYC-209A and WYC-209B exhibited no inhibitory effects at 10 μM toward all five tested CYP enzymes. These findings suggest that potential metabolic toxicity risks of WYC-209 might be negligible.

## Discussion

In this study, we have synthesized and identified a novel small molecule WYC-209 that can inhibit growth of malignant melanoma B16-F1 TRCs in vitro and metastasis of these cells in vivo in immune competent wild-type C57BL/6 mice. Moreover, WYC-209 appears to have little toxic effects in cultured non-cancerous murine 3T3 fibroblasts and in altering the physiological functions and morphologies of organs in wild-type mice. These findings are significant in that it is well known that many existing anti-cancer drugs have encountered drug resistance and relapse in human patients^4,25,26^. Previously, we showed that conventional chemotherapeutic drugs such as doxorubicin and cisplatin were not effective in inducing apoptosis of melanoma TRCs^6^ since ~20–30% of TRCs are not killed even at high concentrations (1 and 100 μM) of these two drugs, suggesting that stem cell-like TRCs might be responsible for drug resistance in malignant melanoma cells. WYC-209 is a new retinoid discovered from the in-house screening of a synthetic retinoid library. Inhibition of B16-F1 TRC growth by WYC-209 is about 10 folds more potent than that of Tazarotene and both enantiomers are highly effective. All-trans retinoic acid is reported to inhibit acute promyelocytic leukemia and breast cancer^27^ via ablation of Pin1, but toxicity^8–11^ and drug resistance^28,29^ of the molecule limit its application in human patients. A recent report shows that an MCL1 inhibitor S63845 is effective against several cancers with reasonable tolerance in mice^30^, but it is not clear at the present time whether this molecule is effective against TRCs. The time-dependent apoptosis of un-selected melanoma cells or melanoma TRCs by WYC-209 reached ~95% 24 h after treatment, much higher than that by Tazarotene (Supplementary Fig. 38), suggesting that WYC-209 is much more effective in killing tumor cells than Tazarotene. One possible role of WYC-209 and its enantiomers is to regulate self-renewing gene *Sox2* and master differentiation gene *Mitf* in B16 cells^6^. We found that *Sox2* expression decreased by ~50% when TRCs were treated with the 10 µM compound, but no change was observed in *Sox2* when TRCs were treated with 0.1 or 1 µM compound. *Mitf* expression did not change when the cells were treated with these compounds at 0.1–10 µM for 24 h (Supplementary Fig. 39). These results suggest that WYC-209 or their two enantiomers did not induce TRC differentiation. The finding that WYC-209 has different impacts on the cells at 1 μM and at 10 μM raises the possibility that WYC-209 binds to protein targets additional to the RARs when the concentration is increased from 1 to 10 μM. Future studies need to be carried out to explore this possibility.

Our finding that the effect of WYC-209 is long-lasting (>5 days) is interesting, since there is no sign of “relapse” or growth of the TRCs in the in vitro cell culture model after single melanoma TRCs were plated back into the soft fibrin gels with fresh medium in the absence of the compound. These results suggest that one might only need to deliver WYC-209 for very limited times to animals or human subjects to abolish the growth potential of malignant melanoma cells. However, whether the long-lasting effect of WYC-209 on melanoma TRCs can be extended to other types of tumorigenic tumor cells in mice or in humans remains to be seen in the future.

The binding assay by surface plasmon resonance (SPR) analysis shows that WYC-209A and WYC-209B acid bind to RARs at nano-molar doses, which are similar to their natural counterpart ATRA, and the competition binding assay has demonstrated that the RAR antagonists could rescue TRC growth inhibited by WYC-209. Furthermore, all human cancer cell lines that we have tested expressed RARs and their expression levels are positively correlated with their sensitivity to WYC-209 (Supplementary Fig. 40). All these data suggest that RARs might be a primary binding target for WYC-209. These findings are supported by the results that when RARα, β, or γ is silenced, the inhibitory effect of WYC-209 on TRC growth is reversed. In addition, consistent with the results that both enantiomers WYC-209A and WYC-209B have similar efficiencies in inhibiting or abrogating growth of melanoma TRCs, RAR antagonists reversed the inhibitory effect of WYC-209A and WYC-209B to a similar extent (Supplementary Fig. 41).

WYC-209A and WYC-209B have only one different configuration at the sulfur atom with different S-O bond orientation (Fig. 1) and hence they are a pair of enantiomers. As the natural ligand of RARs, the terminal acid group at the right end of ATRA is the most important pharmacophore for binding to RARs. With the help of a hydrophobic long chain, the right terminal acid group goes deeply into the hydrophobic binding pocket and binds to Arg269 of RAR residue, while the a,β-ionone ring part of ATRA stays at a relatively lager space near the entrance of the pocket. Tazarotene shares the same binding mode with ATRA except for different hydrophobic chains and left side structures. From their binding information, we speculate that WYC-209A and WYC-209B may adopt similar binding modes as ATRA and Tazarotene, and the chiral sulfur atom at the left side would not affect their binding with RAR. Therefore it is reasonable that the enantiomers WYC-209A and WYC-209B exhibit similar TRC inhibition activities.

In the current study, we treated the mice with WYC-209 once every other day after a 5-day waiting period post tumor cells injection. During the washout assay, the inhibitory effect of WYC-209 on TRC growth appears to be persistent for at least 5 days. Therefore, in the future the protocol for the mice experiments might be optimized for the waiting period before drug addition, intervals between drug injections, and doses. In the future, it will also be of interest to examine the inhibitory effect of WYC-209 on the metastasis of TRCs of human cancer cell lines in nude mice or SCID mice. However, the current study has its strength in that wild-type and immune-competent mice are used as the animal model for mouse melanoma metastasis to the lung since the immune system is known to be important in limiting tumor progression.

In summary, we have synthesized and characterized a novel small molecule, a retinoid, which can effectively inhibit proliferation of malignant TRCs of murine melanoma, human melanoma, human ovarian carcinoma, human breast cancer, and human lung adenocarcinoma in culture. This molecule can inhibit metastasis of murine melanoma TRCs in immune-competent mice with no apparent toxicity or side effects. In the future it will be interesting to see if this novel retinoid can inhibit growth and metastasis of drug-resistant malignant melanoma cells in human subjects.

## Methods

### General synthesis methods

All the reactions were carried out under nitrogen or argon with anhydrous solvents in flame-dried glassware, unless otherwise noted. The chemicals used were reagent grade as supplied, except where noted. For details of the synthetic procedures and the characterization data of compounds, see Supplementary Fig. 1. For ^1^H and ^13^C NMR spectra of the compounds prepared in this study, see Supplementary Figs. 2–5.

### Synthesis of compound **3**

A mixture of 6-ethynyl-4, 4-dimethylthiochroman^31^ (202.3 mg, 1.0 mmol), ethyl 2-chloropyrimidine-5-carboxylate^15^ (155 mg, 0.83 mmol), and Pd(PPh_3_)_2_Cl_2_ (42 mg, 0.06 mmol) was dissolved in DMF (2.0 mL) under argon atmosphere. A mixture of CuI (19 mg, 0.1 mmol) and triethylamine (0.3 mL) in DMF (1.0 mL) was added. The solution was heated to 75 °C for 22 h. The reaction mixture was cooled to room temperature and filtered on a celite pad, followed by washing with ethyl acetate (30 mL). The filtrate was then washed with brine (20 mL) and dried over anhydrous magnesium sulfate. Evaporation of the solvent under vacuum yielded the crude products, which were purified with flash column chromatography to obtain the pure compound **3** (257 mg, 73%). ^1^H NMR (500 MHz, CDCl_3_) δ 9.24 (s, 2H), 7.69 (d, *J* = 1.7 Hz, 1H), 7.33 (dd, *J* = 8.2, 1.8 Hz, 1H), 7.09 (d, *J* = 8.2 Hz, 1H), 4.45 (q, *J* = 7.1 Hz, 1H), 3.11–2.99 (m, 2H), 1.99–1.92 (m, 2H), 1.43 (t, *J* = 7.1 Hz, 3H), 1.33 (s, 6H); ^13^C NMR (126 MHz, CDCl_3_) δ 163.52, 158.44, 155.85, 142.39, 136.48, 131.36, 130.15, 126.83, 121.88, 115.96, 92.65, 87.97, 62.14, 37.09, 33.14, 30.05, 23.43, 14.38. ESI (+)-MS: 353.2 [M + 1]^+^; HRMS-ESI (*m*/*z*) calculated for C_20_H_21_N_2_O_2_S, 353.1318 [M + 1]^+^ and found 353.1317.

### Synthesis of WYC-209

Compound **3** (48 mg, 0.14 mmol) was dissolved in CH_2_Cl_2_ (3.0 mL), and the resulting solution was cooled at 0 °C. *m*-CPBA (34 mg, 0.2 mmol) was added drop-wise under stirring and keeping the reaction temperature below 5 °C. After addition, the mixture was stirred for 1 h at 0 °C and 2 h at room temperature. The *m*-CBA formed was removed by filtration, and the organic phase was washed with a saturated solution of Na_2_S_2_O_5_ (3.0 mL) and with a saturated solution of NaHCO_3_ (3.0 mL), respectively. The organic phase was dried over anhydrous magnesium sulfate and concentrated, the residue was purified with flash column chromatography to provide WYC-209 (43 mg, 83%). ^1^H NMR (500 MHz, CDCl_3_) δ 9.29 (s, 2H), 7.79 (dd, *J* = 10.5, 4.7 Hz, 2H), 7.65 (dd, *J* = 8.1, 1.6 Hz, 1H), 4.47 (q, *J* = 7.1 Hz, 2H), 3.23 (ddd, *J* = 12.7, 10.2, 2.3 Hz, 1H), 3.11 (ddd, *J* = 13.0, 9.2, 2.3 Hz, 1H), 2.43 (ddd, *J* = 15.1, 10.2, 2.3 Hz, 1H), 1.91 (ddd, *J* = 15.1, 9.2, 2.2 Hz, 1H), 1.47 (s, 3 H), 1.44 (t, *J* = 7.2 Hz, 3H), 1.34 (s, 3H); ^13^C NMR (126 MHz, CDCl_3_) δ 163.33, 158.54, 155.32, 145.19, 132.61, 131.06, 130.22, 124.22, 122.62, 89.60, 89.35, 62.32, 43.35, 34.75, 31.36, 31.25, 29.93, 14.39. ESI (+)-MS: 369.4 [M + 1]^+^; HRMS-ESI (*m*/*z*): [M + 1]^+^ calculated for C_20_H_21_N_2_O_3_S, 369.1267 and found 369.1267.

### Synthesis of WYC-209A

Titanium tetraisopropoxide (0.2 mmol, 58 μL) was added rapidly to a solution of *D*-diethyl tartrate (82.7 mg, 0.4 mmol) in 1.0 mL of dichloromethane at 16 °C. After 2.5 min, 10 μL of water was added slowly using a microliter syringe with vigorous stirring for 10 min, followed by quickly cooling to −20 °C for an additional 20 min. The reaction was allowed to take place after rapid addition of compound **3** (69 mg, 0.2 mmol) and cumene hydroperoxide (74 μL, 0.4 mmol). After 3 h the mixture was poured to a solution of citric acid (67 mg) in a mixed solvent of water (4 mL), l, 4-dioxane (2 mL), and diethyl ether (4 mL), and was stirred for 15 min. The aqueous phase was extracted with diethyl ether (3 × 10 mL). The combined organic phases were stirred vigorously with 10 mL of 2 M aqueous K_2_CO_3_ for 0.5 h. The aqueous solution was then extracted with diethyl ether (3 × 10 mL). The combined organic solutions were washed with brine (25 mL), dried over MgSO_4_, and filtered. The enantiomeric excesses (ee) measurement for the enantioselective oxidation was made with the solution, and 75% ee yielded. Then the solution was evaporated under reduced pressure. Flash chromatography of the crude product yielded WYC-209A (43 mg, 84%). The product was recrystallized with ethyl ether for 3 times, and 99% ee product (25 mg) was obtained. All the ee values were measured by chiral HPLC analysis: Agilent 1260 infinity HPLC, Lux Cellulose-1 250 × 4.6 mm column, 20 °C, 254 nm, acetonitrile/H_2_O = 80:20, 0.7 mL min^−1^, retention time 7.81 min. The fractions of sulfoxide were mixed before enantiomeric excesses (ee) measurement. [α]_D_^24^ =  85.6 (c 0.1, CHCl_3_, 99% ee); ^1^H NMR (500 MHz, CDCl_3_) δ 9.29 (s, 2H), 7.82–7.75 (m, 2H), 7.66 (d, *J* = 7.9 Hz, 1H), 4.47 (q, *J* = 7.1 Hz, 2H), 3.24 (t, *J* = 11.1 Hz, 1H), 3.15–3.07 (m, 1H), 2.43 (dd, *J* = 14.0, 10.4 Hz, 1H), 1.92 (dd, *J* = 14.9, 8.8 Hz, 1H), 1.47 (s, 3H), 1.44 (t, *J* = 7.2 Hz, 3H), 1.34 (s, 3H); ^13^C NMR (126 MHz, CDCl_3_) δ 163.33, 158.54, 155.32, 145.19, 132.61, 131.06, 130.22, 124.22, 122.62, 89.60, 89.35, 62.32, 34.75, 31.36, 31.25, 29.93, 14.39; ESI (+)-MS 369.4 [M + 1]^+^; HRMS-ESI (*m*/*z*): [M + 1]^+^ calculated for C_20_H_21_N_2_O_3_S, 369.1267 and found 369.1267.

### Synthesis of WYC-209B

Titanium tetraisopropoxide (0.2 mmol, 58 μL) was added rapidly to a solution of *L*-diethyl tartrate (82.7 mg, 0.4 mmol) in 1.0 mL of dichloromethane at 16 °C. After 2.5 min, 10 μL of water was added slowly using a microliter syringe with vigorous stirring for 10 min, followed by quickly cooling to −20 °C for an additional 20 min. The reaction was allowed to take place after rapid addition of compound **3** (69 mg, 0.2 mmol) and cumene hydroperoxide (74 μL, 0.4 mmol). After 3 h the mixture was poured to the solution of citric acid (67 mg) in 4 mL of water, 2 mL of l, 4-dioxane, and 4 mL of diethyl ether, and was stirred for 15 min. The aqueous phase was extracted with diethyl ether (3 × 10 mL). The combined organic phases were stirred vigorously with 10 mL of 2 M aqueous K_2_CO_3_ for 0.5 h. The aqueous solution was then extracted with diethyl ether (3 × 10 mL). The combined organic solutions were washed with brine (25 mL), dried over MgSO_4_, and filtered. The enantiomeric excesses (ee) measurement for the enantioselective oxidation was made with the solution, and 74% ee yielded. The solution was evaporated under reduced pressure. Flash chromatography of the crude product yielded WYC-209B (43 mg, 84%). The product was recrystallized with ethyl ether for three times, and 99% ee product (23 mg) was obtained. All the ee values were measured by chiral HPLC analysis: Agilent 1260 infinity HPLC, Lux Cellulose-1 250 × 4.6 mm column, 20 °C, 254 nm, acetonitrile/H_2_O = 80:20, 0.7 mL min^−1^, retention time 8.22 min. [α]_D_^24^ =  -85.5 (*c* 0.1, CHCl_3_, 99% ee); ^1^H NMR (500 MHz, CDCl_3_) δ 9.29 (s, 2H), 7.82–7.75 (m, 2H), 7.66 (d, *J* = 7.9 Hz, 1H), 4.47 (q, *J* = 7.1 Hz, 2H), 3.24 (t, *J* = 11.1 Hz, 1H), 3.15–3.07 (m, 1H), 2.43 (dd, *J* = 14.0, 10.4 Hz, 1H), 1.92 (dd, *J* = 14.9, 8.8 Hz, 1H), 1.47 (s, 3H), 1.44 (t, *J* = 7.2 Hz, 3H), 1.34 (s, 3H); ^13^C NMR (126 MHz, CDCl_3_) δ 163.33, 158.54, 155.32, 145.19, 132.61, 131.06, 130.22, 124.22, 122.62, 89.60, 89.35, 62.32, 34.75, 31.36, 31.25, 29.93, 14.39; ESI (+)-MS: 369.4 [M + 1]^+^; HRMS-ESI (*m*/*z*): [M + 1]^+^ calculated for C_20_H_21_N_2_O_3_S, 369.1267 and found 369.1267.

### Animals

Four-week old C57BL/6 female and male mice were obtained from the Center of Medical Experimental Animals of Hubei Province (Wuhan, China). The mice were randomly assigned to the control group or the treated group. As a minimum of eight mice per group was required for having a statistical power, each group had eight mice. The experimentalists were blinded from the expected outcome of the treatment. All animals received humane care in compliance with the Principles of Laboratory Animal Care Formulated by the National Society of Medical Research and the guide for the US National Institutes of Health. The protocol was approved by the Animal Care and Use Committee of Huazhong University of Science and Technology.

### Cell lines and cell culture

Murine melanoma cell line B16-F1, human ovarian cancer cell line A2780, human lung cancer cell line A549, human breast cancer cell line MCF-7, human melanoma cell line MDA-MB-435s, human melanoma cell line A375 and non-cancerous murine fibroblast cell line 3T3 were purchased from the China Center for Type Culture Collection (CCTCC, Wuhan, China), non-cancerous human epidermal cell line HacaT was purchased from Biovector NTCC. In all experiments, cells were randomly allocated to different experimental groups. Since an effective assay for mycoplasma contamination is visual inspection by imaging and DAPI-staining, all cells are counter-stained with DAPI and are constantly monitored for mycoplasma contamination during the course of study. Cells were cultured on rigid dishes with RPMI-1640, Dulbecco’s modified Eagle’s medium or minimum essential medium cell culture medium supplemented with 10% fetal bovine serum (Life Technologies, Carlsbad, CA, USA), and 1% penicillin and streptomycin at 37 °C with 5% CO_2_. Cells were passaged every 3–4 days using TrypLE (Life Technologies). Cell samples were randomly allocated to each well of the culture dishes and randomly chosen for intervention. For all cell culture experiments, at least three independent experiments were performed per condition.

### 3D fibrin gel preparation

Salmon fibrinogen and thrombin were purchased from Reagent Proteins (CA, USA). 3D fibrin gels were prepared as described previously^5,6^. In brief, fibrinogen was diluted into 2 mg mL^−1^ with T7 buffer (pH 7.4, 50 mM Tris, 150 mM NaCl). Cells were detached from 2D rigid dishes and cell density was adjusted to 10^4^ cells per mL. Fibrinogen and cell solution mixture were made by mixing the same volume of fibrinogen solution and cell solution, resulting in 1 mg mL^−1^ fibrinogen and 5000 cells per mL in the mixture fibrin gels. A volume of 50 μL cell/fibrinogen mixture was seeded into each well of 96-well plate and mixed well with pre-added 1 μL thrombin (100 U mL^−1^). The cell culture plate was then incubated in 37 °C cell culture incubator for 10 min. Finally, 200 μL of DMEM medium containing 10% FBS and antibiotics was added.

### Apoptosis assay

Propidium iodide (PI; from Sigma) and 4′, 6-diamidino-2-phenylindole (DAPI; from Sigma) were used to label apoptotic cells and cell nucleus cultured in 3D fibrin gels on Day 5, respectively. Briefly, the cells cultured in 3D fibrin gels were rinsed with pre-warmed PBS and then incubated with 10 µg mL^−1^ PI and 100 ng mL^−1^ DAPI (pre-diluted in normal culture medium) for 1 h. The PI-positive (apoptotic) cells and DAPI-labeled cells were imaged by DMI-6000B fluorescence microscope.

### Compounds washed out assay

Control B16-F1, A2780, A549, MCF-7, MDA-MB-435s, and A375 cells were cultured in 90-Pa fibrin gels to form round colonies and then treated with 0.1, 1, and 10 μM WYC-209, WYC-209A, and WYC-209B or 0.1% DMSO on day 3, which was washed out with pre-warmed PBS on day 4 with fresh medium, and then kept culturing till day 7 or day 9.

### Mice experiments

Four- to six-week-old female and male C57BL/6 mice were used in mice experiment. Mice were randomized into different groups. In metastasis experiment, B16-F1 cell spheroids were selected from 3D 90-Pa fibrin gels and pipetted to single cells. These TRCs were harvested and the cell number was counted under microscopy. The cells were then suspended in PBS with appropriate cell density. Thirty thousand TRCs were intravenously injected into the tail vein of each wild-type C57BL/6 mouse. Five days later, inoculated mice were intravenous implanted with 0.022 mg kg^−1^ WYC-209, 0.22 mg kg^−1^ WYC-209, or 0.1% DMSO every two days. The mice were euthanized and examined for lung tumor formation at day 30. In treated TRCs injected in vivo experiments, B16-F1 cells were seeded into 3D 90-Pa fibrin gels and treated with 10 µM WYC-209 or 0.1% DMSO on day 3, then isolated treated TRCs colonies on day 5 and pipetted into single cell. 30,000 of TRCs were intravenously injected into the tail vein of each wild-type C57BL/6 mouse. No blinding was performed in the mice experiments.

### Histologic evaluation and immunohistochemistry

Lung, liver, spleen, bone, stomach, and brain of C57BL/6 mice were fixed by 4% paraformaldehyde then were embedded in paraffin and cut to ~4 µm thick sections by Thermo FINESSE 325. Organ sections were stained by H&E and slides were evaluated for tumor formation by a veterinary pathologist blinded to sample identity.

### Flow cytometry

For Annexin-V and PI staining to quantify cell apoptosis, treated TRCs were isolated from 3D fibrin gel by using 5 mg mL^−1^ Dispase II (Sigma), cells were labeled with FITC-conjugated Annexin-V and PI according to the manufacturer’s instruction (Biolegend). Data were acquired on a BD Cantoll (BD biosciences) and analyzed with FlowJo software. For BrdU staining to quantify cell proliferation, cells were labeled with FITC-conjugated anti-BrdU antibody according to the manufacturer’s instruction (FITC BrdU Flow Kit, BD Biosciences) and then data were acquired on an Accuri C6 (BD Biosciences) and analyzed with BD Accuri C6 software.

### Western blotting assay

To quantify the expression levels of RARα, RARβ, RARγ, caspase-3, cleaved caspase-3, and cleaved PARP, cells were lysed with RIPA lysis buffer. Each sample were separated by 8–15% SDS-PAGE, blocked with 5% BSA over night at 4 °C, and incubated with primary antibodies to RARα (1:1000, Abcam, ab28767), RARβ (1:500, Abcam, ab53161), RARγ (1:500, Abcam, ab97569), caspase-3 (1:1000, Cell Signal, 9662S), cleaved caspase-3 (1:1000, Cell Signal, 9664T), or cleaved PARP (1:1000, Cell Signal, 9548T) overnight at 4 °C. Primary antibodies were detected with mouse anti-Rabbit IgG-HRP (1:1000, Santa Cruz, sc-2357), donkey anti-goat IgG-HRP (1:1000, Santa Cruze, sc-2020), or anti-Mouse IgG-HRP (1:1000, Santa Cruz, sc-2005).

### Growth inhibition calculation

The growth inhibition of human or murine TRCs by WYC-209 were calculated by the formula below: Growth inhibition = 100% × [(Colony size_DMSO at Day5_ − Colony size_DMSO at Day 0_) − (Colony size_WYC-209 at day5_ − Colony size_WYC-209 at Day0_)]/(Colony size_DMSO at Day5_ − Colony size_DMSO at Day 0_).

### MTT cell proliferation assay

Cell proliferation rate was measured by using MTT (3-(4,5- dimethylthiazol-2-yl)-2,5-diphenyltetrazolium bromide) colorimetric assay (abcam). Cells were seeded in 3D soft fibrin gel in 96-well microplates at a density to maintain control (untreated) cells in an exponential phase of growth during the entire experiment. Cells were incubated with various concentrations of compounds for each time point followed by incubation with 50 µL MTT reagent (abcam) for 3 h at 37 °C. After incubation, 150 µL MTT solvent (abcam) was added and absorbance was measured at 570 nm. All experiments were repeated at least three times. The percentage of viable cells was calculated and averaged for each well: percent growth = (OD-treated cells/OD control cells) × 100, cell proliferation at each time point is normalized to base line cell survival at the time of initial compound treatment (day 0).

### *K*_*d*_ quantitation using surface plasmon resonance

Surface plasmon resonance (SPR) was carried out by Shanghai Medicilon Inc, using the Biacore 8K equipment. Human protein RARα (ab82196), RARβ (ab82202), and RARγ (ab81922) were purchased from Abcam (US) Ltd. Proteins were immobilized by amine coupling. Experiments were performed at 25 °C at 30 μL min^−1^ with high-performance injection and the data collected at 10 Hz. Both association and dissociation times were 120 s. Prime into run buffer contains PBS with 0.05% P20 and 5% DMSO. Immobilized proteins via the amine coupling procedure were diluted to 50 μg mL^−1^ in 10 mM sodium acetate (pH 5.0; 420 s injection time at 10 μL min^−1^). Compound binding was plotted in a 9-point dose–response curve with no compound as the reference. Highest concentration was 10 μM for the compounds. Final DMSO concentration was 5.0% for all compounds. Executed runs included three startup injections and a 4-point solvent correction at the beginning and the end of the run.

### RNA interference

Cells were transfected with siRNA using Lipofectamine 2000 (invitrogen) following the manufacturer’s protocol. Silencer Negative Control No. 1 siRNA (Invitrogen, AM4611) was used a negative control in RNA interference experiments. The sequences of siRNAs are: UUAAAAAAAAAAAAUGGUUTT for *mouse RARα #1*; UAAAUGUGCUUAAAAUGAATT for *mouse RARα #2*; UAUCUGGGGAUUGGUACGCTT for *mouse RARβ #1*; UAUAUGAACAUAGAAAGCATT for *mouse RARβ #2*; CUAAUAAAUAAAUAGAGGCTT for *mouse RARγ #1*; ACUUUGGCAAAAUAACGAGTT for *mouse RARγ #2*; GGAUAGUGUUUCUAAGGAATT for *mouse caspase 3 #1*; GCCAACCUCAGAGAGACAUTT for *mouse caspase 3 #2*; GGGAUCUAUCUGGACAGUATT for *mouse caspase 3 #3*; CACAGCACCUGGUUAUUAUTT for *human caspase 3 #1*; CCCUGGACAACAGUUAUAATT for *human caspase 3 #2*; CCGACAAGCUUGAAUUUAUTT for *human caspase 3 #3*.

### Real-time RT analysis

Total mRNA was isolated from the cells using the Trizol reagent according to the supplier’s instruction (Invitrogen). Reverse transcription (RT) was performed using the TransScript First-strand cDNA Synthesis SuperMix (TransGen), according to the manufacturer’s protocol. Real-time RT-PCR was performed using *GoTaq* qPCR Master Mix (Promega). The data were normalized against mouse glyceraldehyde 3-phosphate dehydrogenase. The primer sequences are as follows: mouse GAPDH (F: 5′-AGGTCGGTGTGAACGGATTTG-3′, R: 5′-TGTAGACCATGTAGTTGAGGTCA-3′); mouse *RARα* (F: 5′-TCAGTGCCATCTGCCTCATCT-3′, R: 5′-ATGCTCCGAAGGTCTGTGATCT-3′); mouse *RARβ* (F: 5′-CTGCTTGCCTGGACATCCTAAT-3′, R: 5′-CAGTCTCGGTGTCATCCATCTC-3′); mouse *RARγ* (F: 5′-AATGCTGGCTTCGGTCCTCT-3′, R:5′-CCTGGCGGTCTCCACAGATTA-3′); mouse *Sox2* (F: 5′-GCAGTACAACTCCATGACCA-3′, R: 5′-CCTCGGACTTGACCACAGA-3′); mouse *Mitf* (F: 5′-TACCAACAACCTCGGCACCAT-3′, R: 5′-GCTCCTGGCGACACTGATGA-3′).

### Functional hERG blocking assay for WYC-209A and WYC-209B

The hERG blocking assays for WYC-209A and WYC-209B were evaluated in the CHO cell line stably expressing hERG channels (Sophion Bioscience, Denmark) using automated conventional manual patch clamp (HEKA EPC-10, Germany), while the cisapride was used as the positive control. Whole-cell tail currents of hERG channels were tested during the experiments at six doses (30, 10, 3, 1, 0.3, and 0.1 µM); *n* ≥ 2 for each dose. The IC_50_ values were determined with XLFit or Graphpad Prism curve fitting software.

### Cytochromes enzyme inhibition assay for WYC-209A and WYC-209B

To evaluate the inhibitive potential of WYC-209A and WYC-209B on human liver microsomes, five representative metabolic enzymes, including cytochromes P450 (CYP) 1A2, 2C9, 2C19, 2D6, and 3A4 were selected and tested with each isomer using well-established specific inhibiting systems for each CYP enzyme. The substrate drugs used for CYP 1A2, 2C9, 2C19, 2D6, and 3A4 were phenacetin (30 µM), diclofenac (10 µM), *S*-mephenytoin (35 µM), bufuralol (10 µM), and testosterone (80 µM), respectively, while the control inhibitors for CYP 1A2, 2C9, 2C19, 2D6, and 3A4 were α-naphthoflavone (0.3 µM), sulfaphenazole (10 µM), omeprazole (100 µM), quinidine (2.5 µM), and ketoconazole (2.5 µM), respectively. After incubation for 10, 10, 45, 10, and 5 min, respectively, the metabolic products of these substrates were analyzed by liquid chromatography (LC)-mass spectrometry (MS). All data were obtained from duplicate assays.

### Statistical analysis

Graphpad Prism software was used for generating Kaplan–Meier animal survival plots of 0.1% DMSO and compounds treated mice and performing statistical analysis (using a log-rank test (Mantel–Cox)). Mice lung metastasis experiments were analyzed by Fisher’s exact test. All other experimental data were analyzed using a two-tailed Student’s *t*-test; when multiple comparisons were carried out within a set of experiments, Bonferroni correction was also performed.

### Data availability

The X-ray crystal structures of WYC-209A and WYC-209B are stored in Cambridge Crystallographic Data Centre (CCDC) with the accession codes CCDC 1822478 and CCDC 1822479. Other data that support the findings of this study are available from the corresponding author upon reasonable request.

## Electronic supplementary material

Supplementary Information

## References

[1] Hanahan D, Weinberg RA (2011). Hallmarks of cancer: the next generation. Cell.

[2] Holohan C, Van Schaeybroeck S, Longley DB, Johnston PG (2013). Cancer drug resistance: an evolving paradigm. Nat. Rev. Cancer.

[3] Visvader JE, Lindeman GJ (2012). Cancer stem cells: current status and evolving complexities. Cell. Stem. Cell..

[4] Dean M, Fojo T, Bates S (2005). Tumour stem cells and drug resistance. Nat. Rev. Cancer.

[5] Liu J (2012). Soft fibrin gels promote selection and growth of tumorigenic cells. Nat. Mater..

[6] Tan Y (2014). Matrix softness regulates plasticity of tumour-repopulating cells via H3K9 demethylation and Sox2 expression. Nat. Commun..

[7] Chen J (2016). Efficient extravasation of tumor-repopulating cells depends on cell deformability. Sci. Rep..

[8] Penniston KL, Tanumihardjo SA (2006). The acute and chronic toxic effects of vitamin A. Am. J. Clin. Nutr..

[9] Tallman MS (1997). All-trans-retinoic acid in acute promyelocytic leukemia. New Engl. J. Med..

[10] Shih MYS (2009). Retinol esterification by DGAT1 is essential for retinoid homeostasis in murine skin. J. Biol. Chem..

[11] Nau H (1993). Embryotoxicity and teratogenicity of topical retinoic acid. Skin. Pharmacol..

[12] Tang XH, Gudas LJ (2011). Retinoids, retinoic acid receptors, and cancer. Annu. Rev. Pathol. Mech. Dis..

[13] Maire AL (2012). Retinoid receptors and therapeutic applications of RAR/RXR modulators. Curr. Top. Med. Chem..

[14] Chandraratna, R. A. Disubstituted acetylenes bearing heteroaromatic and heterobicyclic groups having retinoid like activity. US Patent #5,089,509 (1989).

[15] Ohta K (2000). Retinoidal pyrimidinecarboxylic acids, unexpected diaza substituent effects in retinobenzoic acids. Chem. Pharm. Bull..

[16] Boehm MF (1995). Design and synthesis of potent retinoid X receptor selective ligands that induce apoptosis in leukemia cells. J. Med. Chem..

[17] Brunel JM (1995). Highly enantioselective oxidation of sulfides mediated by a chiral titanium complex. J. Org. Chem..

[18] Vasudevan J, Chandraratna R (2004). Rational design of tazarotene. J. Am. Aacd. Dermatol..

[19] Shistik G, Prakash AV, Fenske NA, Glass LF (2007). Treatment of locally metastatic melanoma: a novel approach. J. Drugs Dermat..

[20] Valastyan S, Weinberg RA (2011). Tumor metastasis: molecular insights and evolving paradigms. Cell.

[21] Widschwendter M (2000). Methylation and silencing of the retinoic acid receptor-beta 2 gene in breast cancer. J. Natl. Cancer I.

[22] Goodyer P, Dehbi M, Torban E, Bruening W, Pelletier J (1995). Repression of the retinoic acid receptor-alpha gene by the Wilms’ tumor suppressor gene product, wt1. Oncogene.

[23] Devarajan E (2002). Down-regulation of caspase 3 in breast cancer: a possible mechanism for chemoresistance. Oncogene.

[24] Enari M, Talanian RV, Wong WW, Nagata S (1996). Sequential activation of ICE like and CPP32 like proteases during FAS mediated apoptosis. Nature.

[25] Visvader JE, Lindeman GJ (2008). Cancer stem cells in solid tumours: accumulating evidence and unresolved questions. Nat. Rev. Cancer.

[26] Vermeulen L, de Sousa e Melo F, Richel DJ, Medema JP (2012). The developing cancer stem-cell model: clinical challenges and opportunities. Lancet Oncol..

[27] Wei S (2015). Active Pin1 is a key target of all-trans retinoic acid in acute promyelocytic leukemia and breast cancer. Nat. Med..

[28] Lehmann-Che J, Bally C, de Thé H (2014). Therapy resistance in APL. N. Engl. J. Med..

[29] de Thé H (2018). Differentiation therapy revisited. Nat. Rev. Cancer.

[30] Kotschy A (2016). The MCL1 inhibitor S63845 is tolerable and effective in diverse cancer models. Nature.

[31] Kumar, B. V. S. *et al*. Process for the preparation of 4, 4-dimethyl-6-ethynylthiochroman. US Patent #6,963,002 (2004).

