# Supplementary material for: Inhibition of cancer stem cell like cells by a synthetic retinoid
Source: Nat Commun. 2018 Apr 11;9:1406. doi: 10.1038/s41467-018-03877-7 (PMC5895803; doi:10.1038/s41467-018-03877-7)
Supplement: Supplementary file 1 — Supplementary Information [file 41467_2018_3877_MOESM1_ESM.pdf]

Supplementary Information

Inhibition of cancer stem cell like cells by a synthetic retinoid

Chen *et al*.

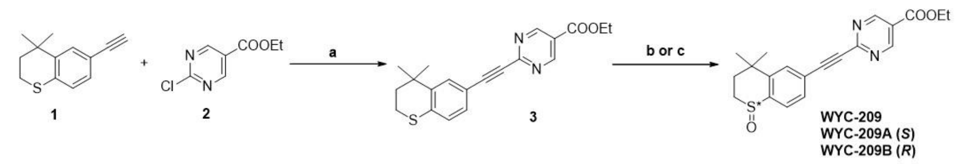

Supplementary Figure 1. Synthetic route for WYC-209 and WYC-209A and WYC-209B. (a) $(PPh_3)_2PdCl_2$, CuI, Et_3N, DMF, 75 ^oC, 73%; (b) *m*-CPBA, DCM, 0 ^oC to rt, 83%; (c) *D/L*-DET, Ti(*i*-PrO)$_4$, cumene hydroperoxide, H_2O, DCM, 75% ee 84% yield for WYC-209A, and 74% ee and 84% yield for WYC-209B, respectively.

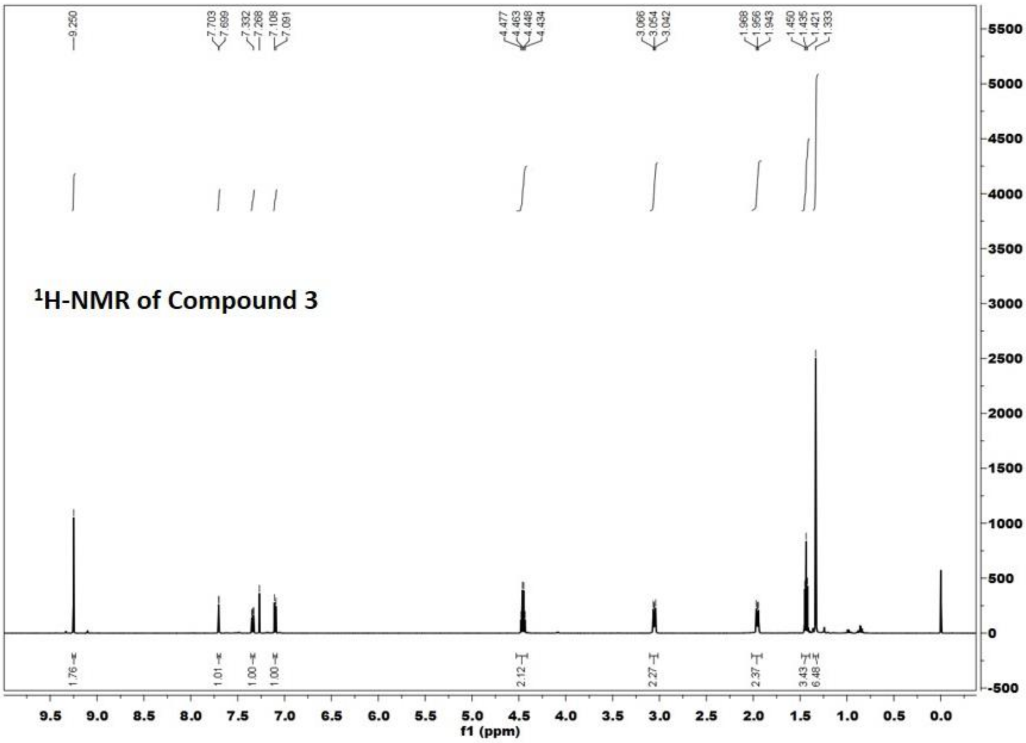

Supplementary Figure 2. ^{1}H-NMR of Compound 3.

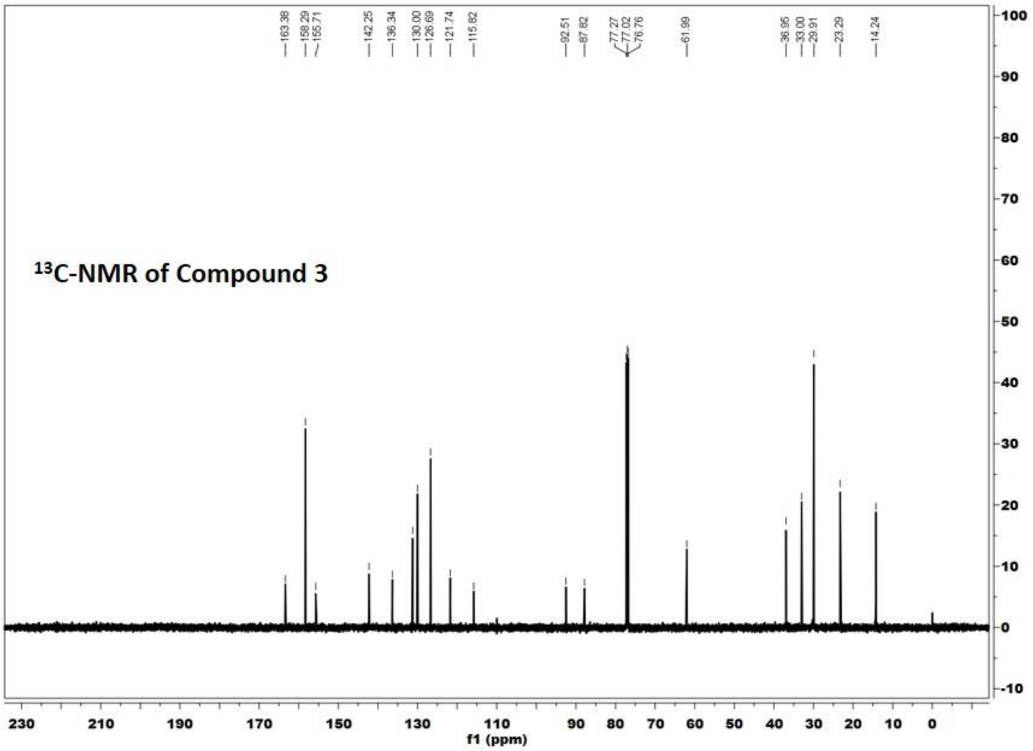

Supplementary Figure 3. ^{13}C-NMR of Compound 3.

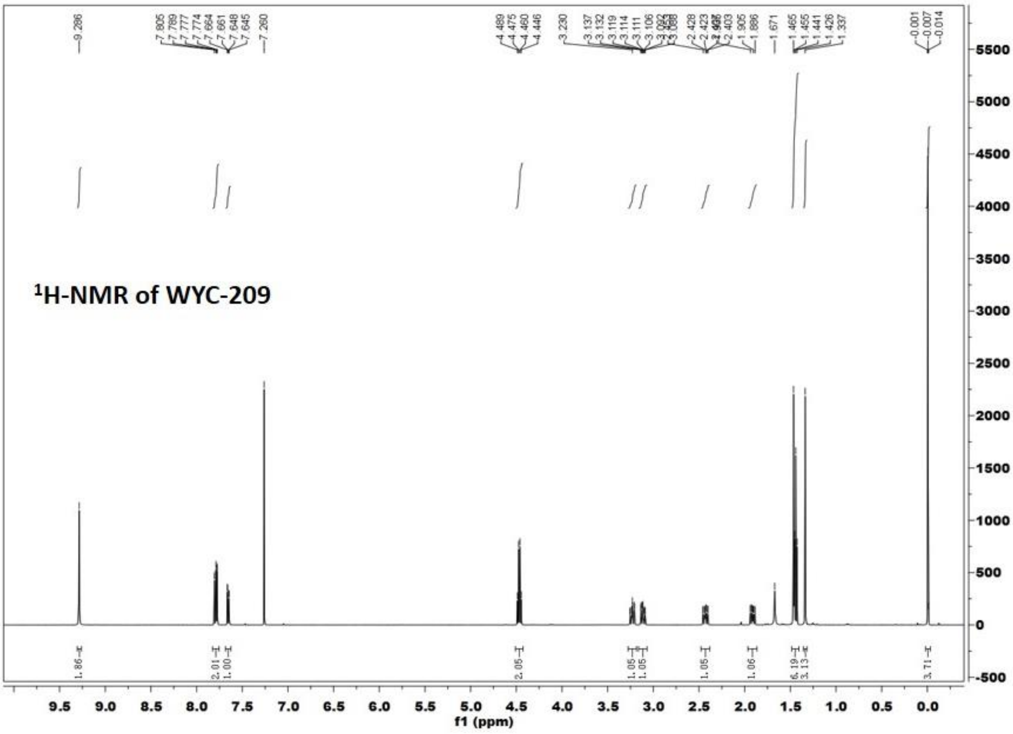

Supplementary Figure 4. ^{1}H-NMR of WYC-209.

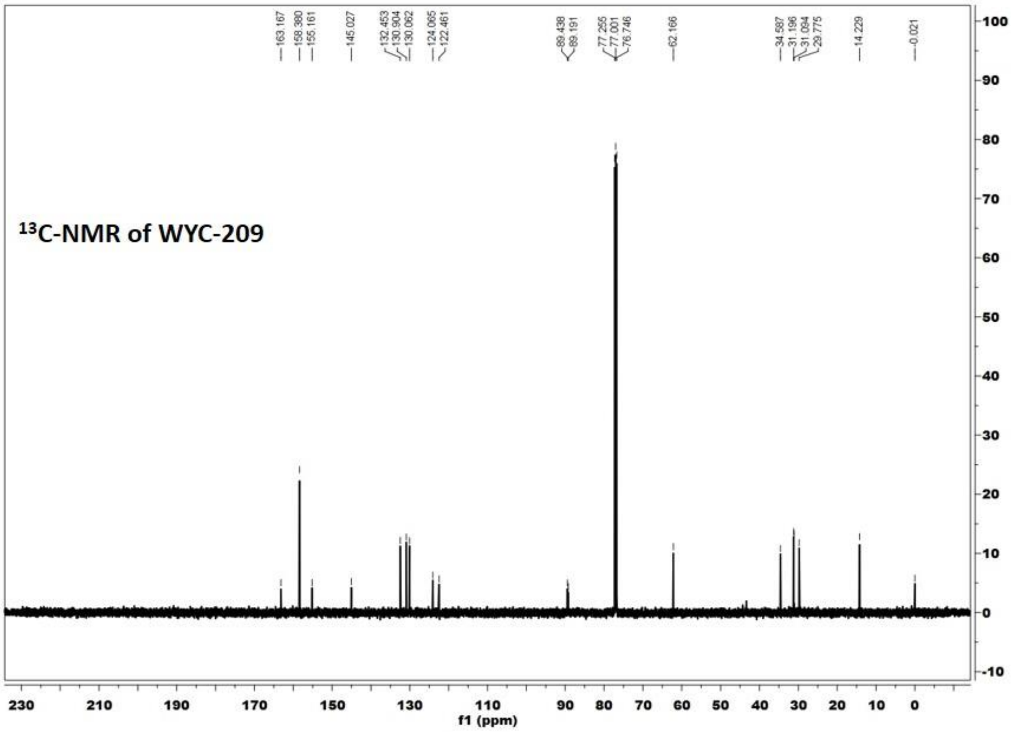

Supplementary Figure 5. ^{13}C-NMR of WYC-209.

Chromatogram and Results

Injection Details

Injection Name:	SFC-+- PC1 A8W2 254 0.7 20	*Run Time (min):*	18.29
Vial Number:	RA1	*Injection Volume:*	2.00
Injection Type:	Unknown	*Channel:*	UV_VIS_1
Calibration Level:		*Wavelength:*	254.0
Instrument Method:	20160223-DAD2	*Bandwidth:*	4
Processing Method:	20160223	*Dilution Factor:*	1.0000
Injection Date/Time:	###############	*Sample Weight:*	1.0000

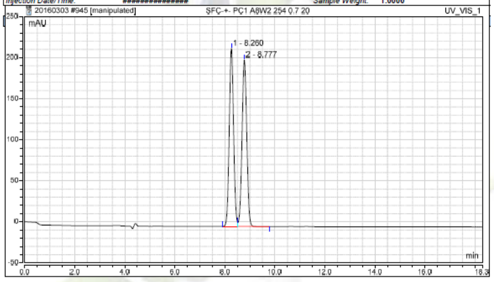

Integration Results

No.	Retention Time min	Area mAU*min	Height mAU	Relative Area %
1	8.260	44.107	217.152	49.94
2	8.777	44.217	202.811	50.06
Total:		88.324	419.963	100.00

Chromatogram and Results

Injection Details

Injection Name:	SFC-209-1 PC1 A8W2 254 0.7 20	*Run Time (min):*	13.00
Vial Number:	RA2	*Injection Volume:*	2.00
Injection Type:	Unknown	*Channel:*	UV_VIS_1
Calibration Level:		*Wavelength:*	254.0
Instrument Method:	20160223-DAD2	*Bandwidth:*	4
Processing Method:	20160223	*Dilution Factor:*	1.0000
Injection Date/Time:	###############	*Sample Weight:*	1.0000

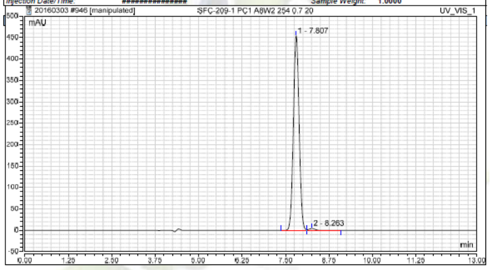

Integration Results

No.	Retention Time min	Area mAU*min	Height mAU	Relative Area %
1	7.807	86.802	454.905	98.93
2	8.263	0.939	4.459	1.07
Total:		87.740	459.364	100.00

Chromatogram and Results

Injection Details

Injection Name:	SFC-209-2 PC1 A8W2 254 0.7 20	*Run Time (min):*	13.00
Vial Number:	RA3	*Injection Volume:*	2.00
Injection Type:	Unknown	*Channel:*	UV_VIS_1
Calibration Level:		*Wavelength:*	254.0
Instrument Method:	20160223-DAD2	*Bandwidth:*	4
Processing Method:	20160223	*Dilution Factor:*	1.0000
Injection Date/Time:	###############	*Sample Weight:*	1.0000

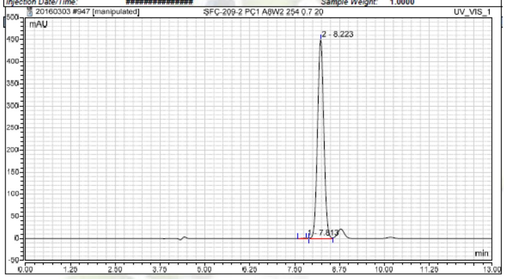

Integration Results

No.	Retention Time min	Area mAU*min	Height mAU	Relative Area %
1	7.813	0.142	0.836	0.16
2	8.223	91.104	450.071	99.84
Total:		91.246	450.907	100.00

Supplementary Figure 6. Chiral HPLC spectra of WYC-209, WYC-209A, and WYC-209B.

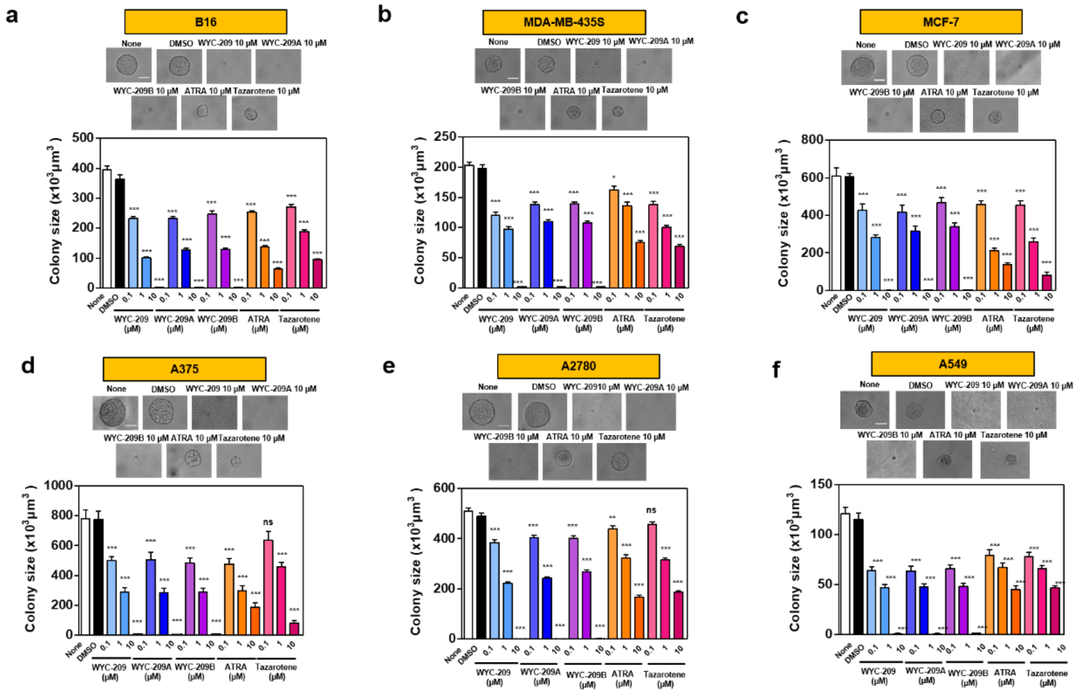

Supplementary Figure 7. WYC-209 has more inhibitory effects on TRCs than ATRA and Tazarotene. Murine melanoma B16-F1 (**a**), human melanoma MDA-MB-435s (**b**), human breast cancer MCF-7 (**c**), human malignant melanoma A375 (**d**), human ovarian cancer A2780 (**e**) and human lung cancer A549 (**f**) were cultured in 90-Pa fibrin gels and treated with 0.1, 1, or 10 μM WYC-209, WYC-209A, WYC-209B, ATRA, or Tazarotene at day 0. **None**: cell medium only, **DMSO**: medium with 0.1% DMSO. Colony size of TRCs were quantified on day 7 for different human cancer cell lines and on day 5 for murine B16-F1. Mean ± s.e.m.; $^{*}P$<0.05; ** P<0.01; *** P<0.001 (Student's t-test); n=15 samples; 3 independent experiments. Bright field images were representative images of colonies in (**a**) on day 5 and in (**b-f**) on day 7 respectively. Scale bars, 50 μm.

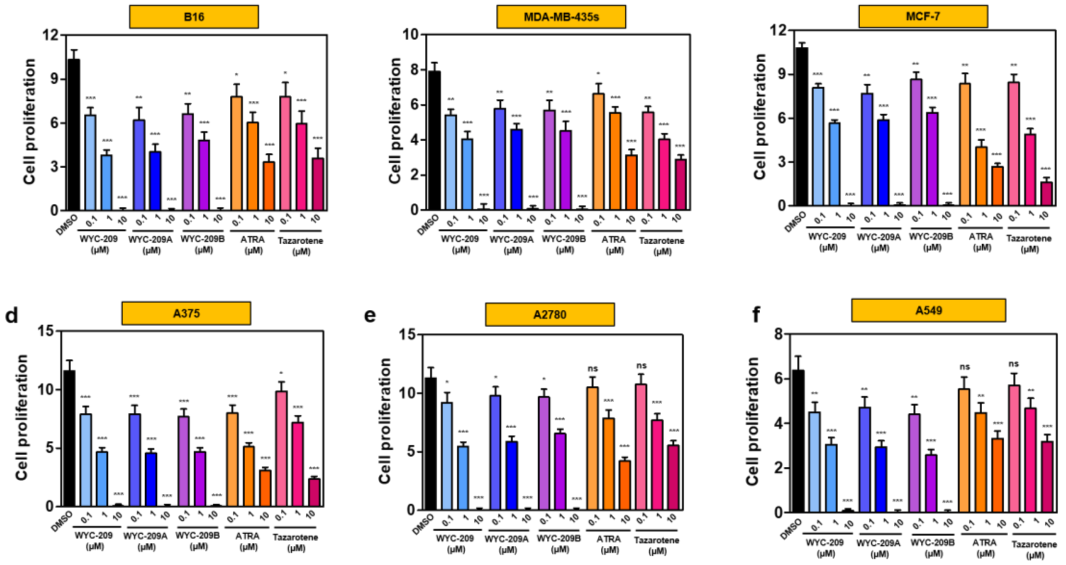

Supplementary Figure 8. WYC-209 abrogates proliferation of TRCs. Murine melanoma B16-F1 (**a**), human melanoma MDA-MB-435s (**b**), human breast cancer MCF-7 (**c**), human malignant melanoma A375 (**d**), human ovarian cancer A2780 (**e**) and human lung cancer A549 (**f**) were cultured in 90-Pa fibrin gels and treated with 0.1, 1, or 10 μM WYC-209, WYC-209A, WYC-209B, ATRA and Tazarotene at day 0. **DMSO**: medium with 0.1% DMSO. Cell proliferation rate was quantified by using MTT to record cell survival on day 7 of five different human cancer cell lines and on day 5 of B16-F1, and data are normalized to baseline cell survival at the time of initial drug treatment (day 0). Mean ± s.e.m.; ns, not significantly different; $^{*}P<0.05$; $^{**}P<0.01$; $^{***}P<0.001$ (Student's t-test); n=3 independent experiments.

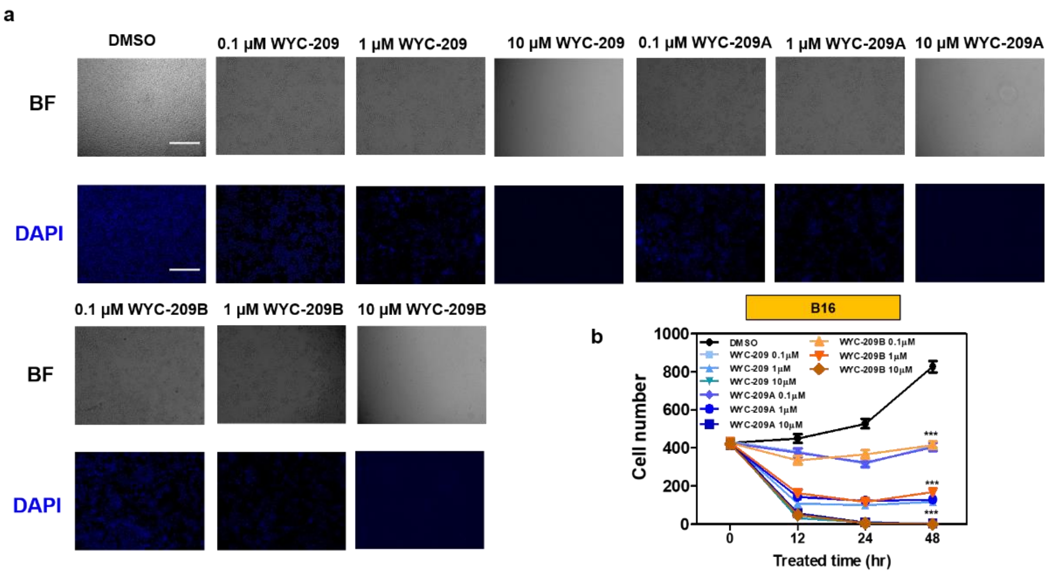

Supplementary Figure 9. WYC-209 detaches adherent melanoma cells on rigid plastic. Murine melanoma cell line B16-F1 were cultured on rigid plastics and treated with 10 µM WYC-209 and observed for 48 hours. Cell nucleus were stained with DAPI. **DMSO**: medium containing 0.1% DMSO. (**a**) Representative images of cells stained with DAPI 48 hrs after treatment. Scale bars, 500 µm. (**b**) Quantitation of cell numbers per view field of B16-F1 treated with WYC-209. Mean ± s.e.m.; n=5 samples; 3 independent experiments; *** P<0.001 (Student's t-test).

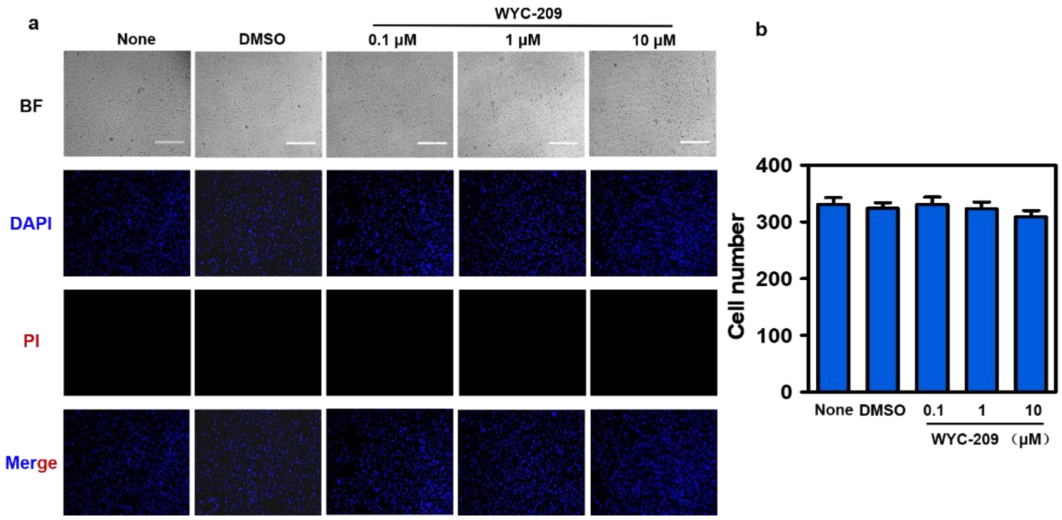

Supplementary Figure 10. WYC-209 has little toxicity on non-cancerous cells. Murine fibroblast cell line 3T3 cells were cultured on rigid plastic and treated with 0.1, 1, or 10 μM WYC-209 at the time of plating and observed for 18 hrs. Cell nucleus were stained with DAPI and apoptotic cells were assayed with PI. **None**: medium only; **DMSO**: medium containing 0.1% DMSO. (**a**) Representative images of cells at hour 18. (**b**) Quantitation of cell numbers per view field at hour 18. Mean ± s.e.m.; n= 5 randomly chosen view-fields; 3 separate experiments; no significant differences were observed between groups (Student's t-test). Scale bars, 500 μm.

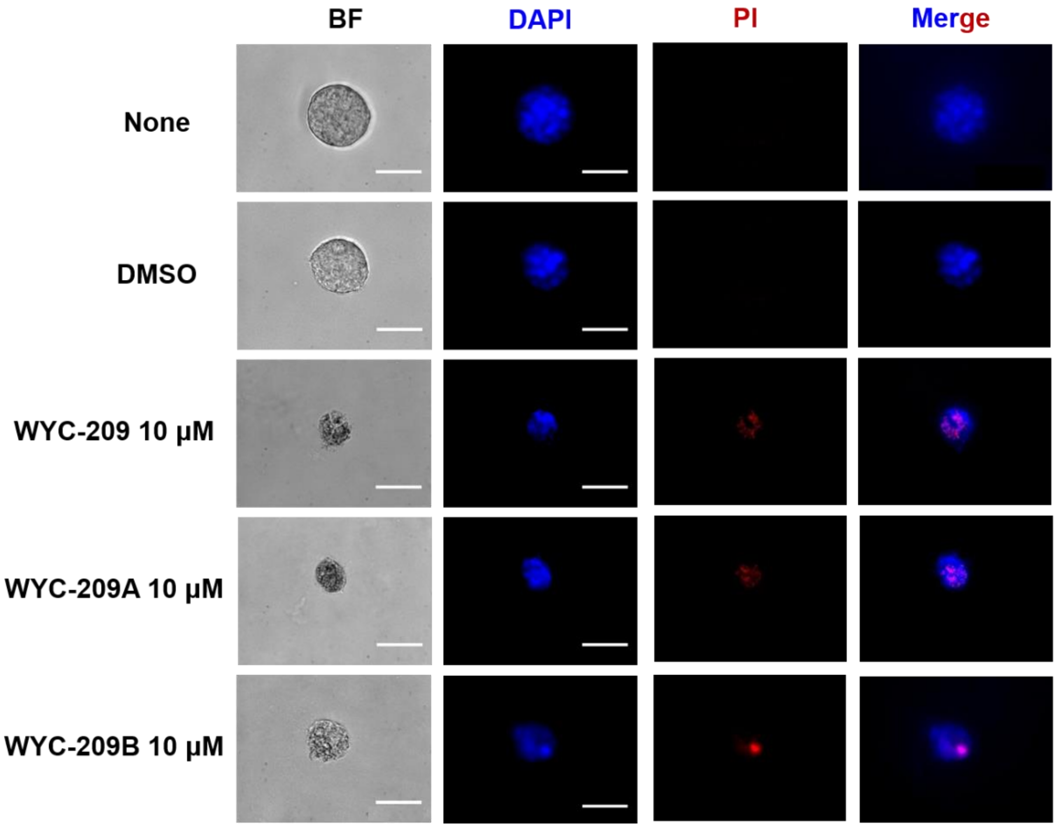

Supplementary Figure 11. WYC-209 (A/B) induces TRC apoptosis. Control B16-F1 cells were cultured in 90-Pa fibrin gels for 3 days till colony formed and treated with **None** (medium without drugs), **DMSO** (medium with 0.1% DMSO), 10 μM WYC-209, 10 μM WYC-209A, or 10 μM WYC-209B, respectively. At day 5, cell nucleus was stained with DAPI and cell apoptosis was assayed with Propidium Iodide (PI). In the figure, PI-positive cells represent apoptotic cells. Scale bars, 50 μm.

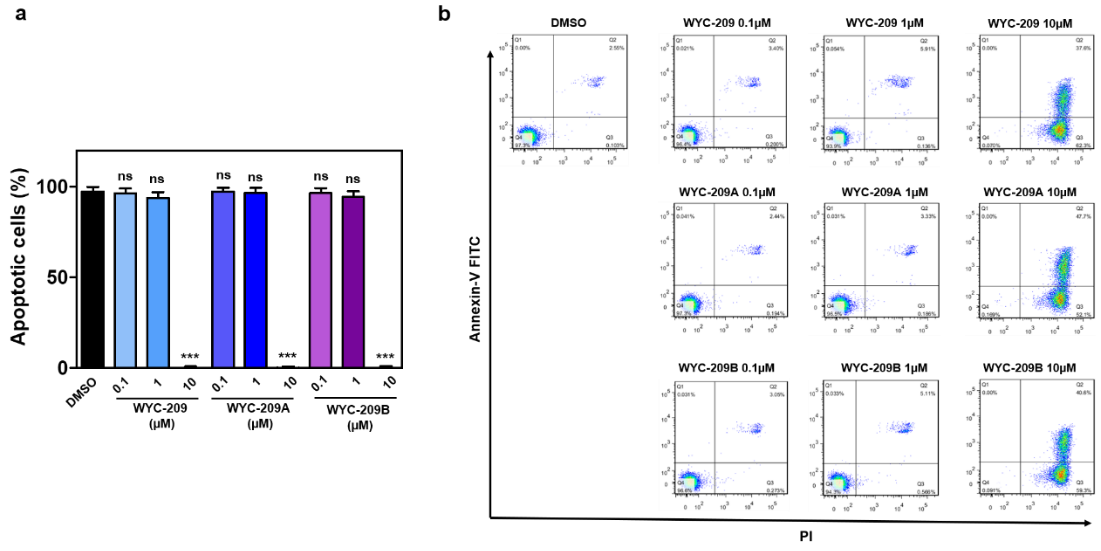

Supplementary Figure 12. WYC-209 induces TRC apoptosis at 10 μM. (**a**) Murine melanoma B16-F1 cells were seeded into 3D soft fibrin gels and then treated with 0.1% DMSO, 0.1, 1, or 10 μM WYC-209, WYC-209A, or WYC-209B on day 4. The cells were collected after 24 hours and stained with FITC-conjugated Annexin-V and PI for apoptotic detection by flow cytometry. Apoptotic cells (%) = 100% - (Annexin-V^{-}PI^{-}) %. Mean ± s.e.m.; 3 separate experiments; *** *P*<0.001 (Student's t-test). (**b**) Original images of flow cytometry of (**a**).

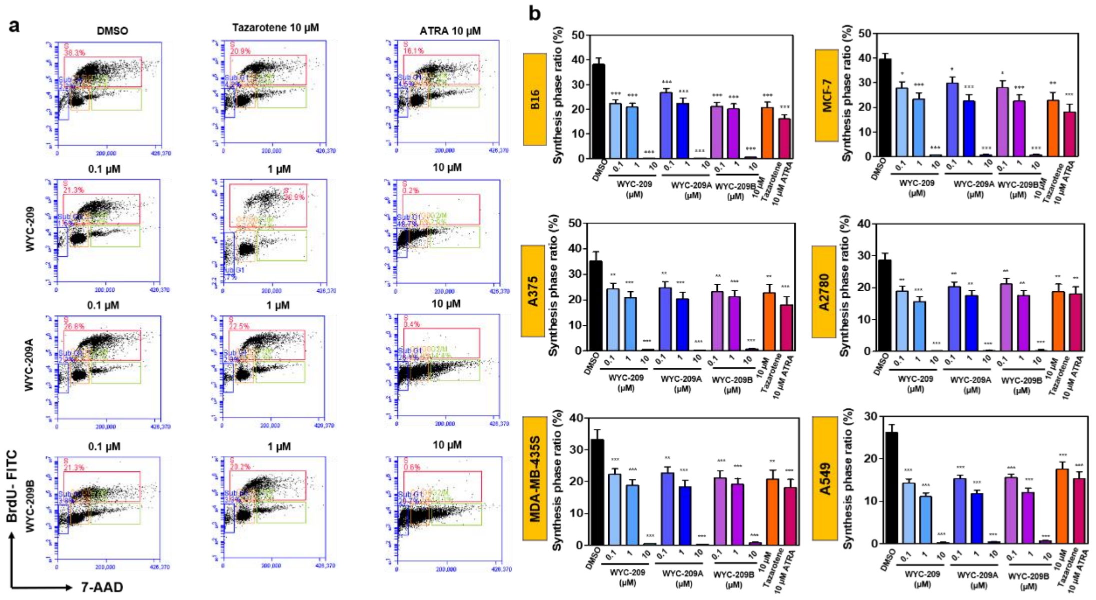

Supplementary Figure 13. WYC-209 blocks S phase of TRCs. Different cancer cell lines cultured in 3D soft fibrin gel on day 0, then treated with 0.1, 1, or 10 μM WYC-209, WYC-209A, WYC-209B on day 4, isolated cells after 24 hours and labeled with FITC-conjugated anti-BrdU antibody for cell cycle analysis, 10 μM ATRA and Tazarotne as positive controls. (**a**) Representative images of flow cytometry in different conditions. (**b**) Quantification of S phase ratio in different conditions. Mean ± s.e.m.; n=3 independent experiments; * P<0.05; ** P<0.01; *** P<0.001 (Student's t-test).

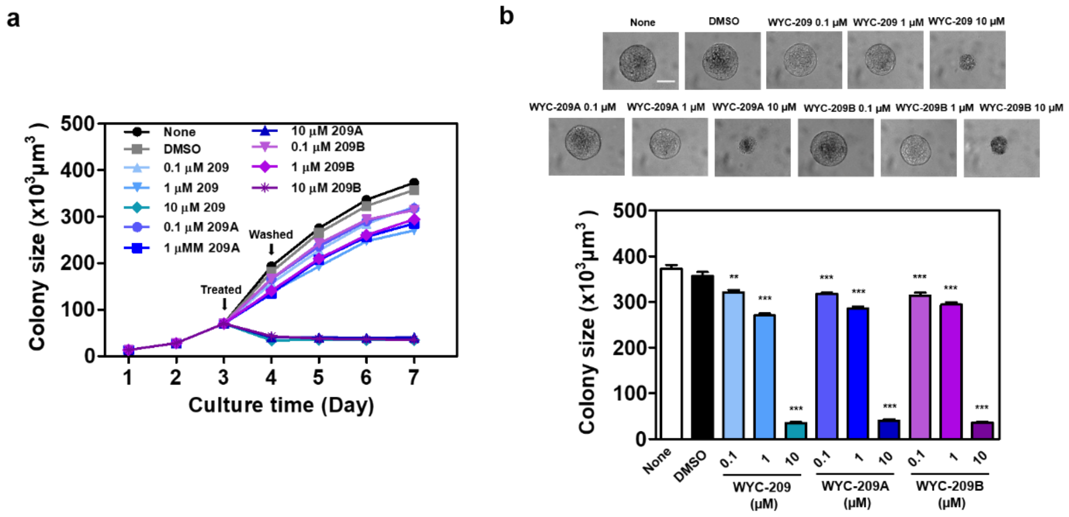

Supplementary Figure 14. Inhibition of tumor cell growth by WYC-209 after washout. Murine melanoma cells were cultured in 90-Pa fibrin gels for 3 days and then treated with medium alone (**None**), **DMSO** (0.1% DMSO-containing medium), 0.1, 1 or 10 μM WYC-209, WYC-209A, or WYC-209B, and washed out on day 4. (**a**) Quantitative data of colony sizes from day 1 to day 7. (**b**) Quantitative data of colony sizes on day 7. Mean ± s.e.m.; n= 15 samples; 3 separate experiments; ** *P*<0.01; *** *P*<0.001 (Student's t-test). Bright field images were representative images of colonies on day 7. Scale bar, 50 μm.

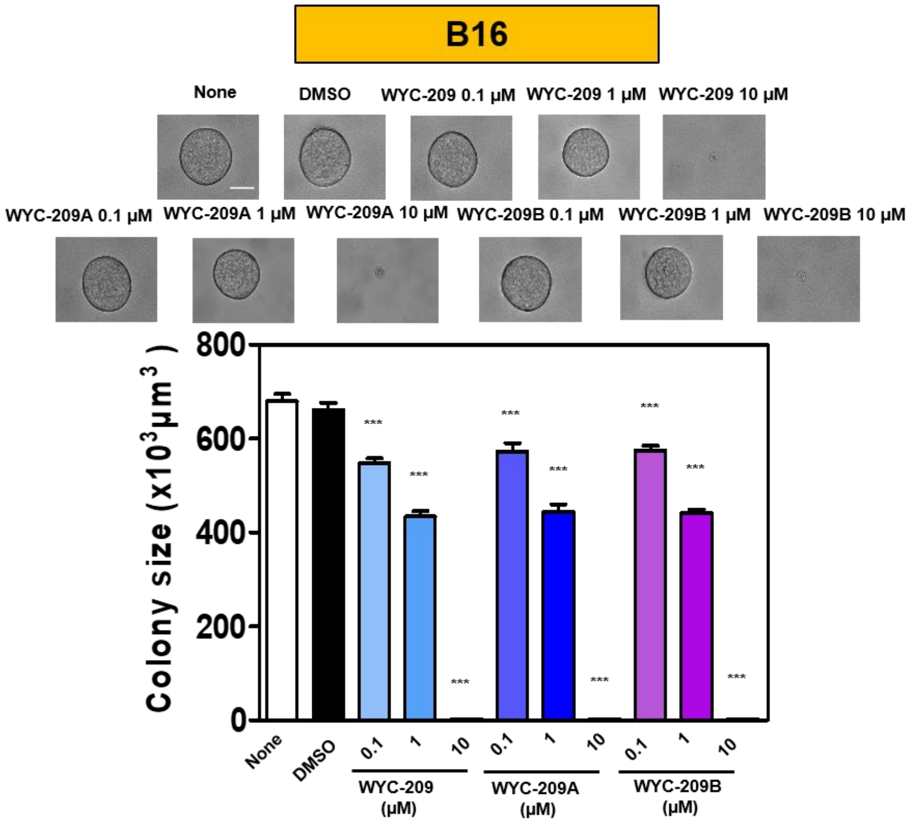

Supplementary Figure 15. WYC-209 abrogates colony growth of murine TRCs after re-plating. DMSO: Control B16-F1 cells cultured in 90-Pa 3D fibrin gels and treated with 0.1% DMSO and compounds on day 0 for 5 days, then re-plated into 90-Pa 3D fibrin gels as single individual cells for 5 days without treatment. **0.1 μM, 1 μM, or 10 μM WYC-209, WYC-209A, or WYC-209B:** Control B16-F1 cells, cultured in 90-Pa 3D fibrin gels and pretreated with WYC-209, WYC-209A, or WYC-209B on day 0 with various concentrations for 5 days. Then after washing out WYC-209, WYC-209A and WYC-209B, the cells were re-plated into 90-Pa 3D fibrin gels for 5 days without any treatment. Representative images of colonies on day 5 (all colonies started as a single cell on day 0); Scale bar, 50 μm. Mean ± s.e.m.; n= 15 samples; 3 separate experiments. *** P<0.001 (Student's t-test). Bright field images were representative images of colonies on day 5 after re-plating. Scale bar, 50 μm.

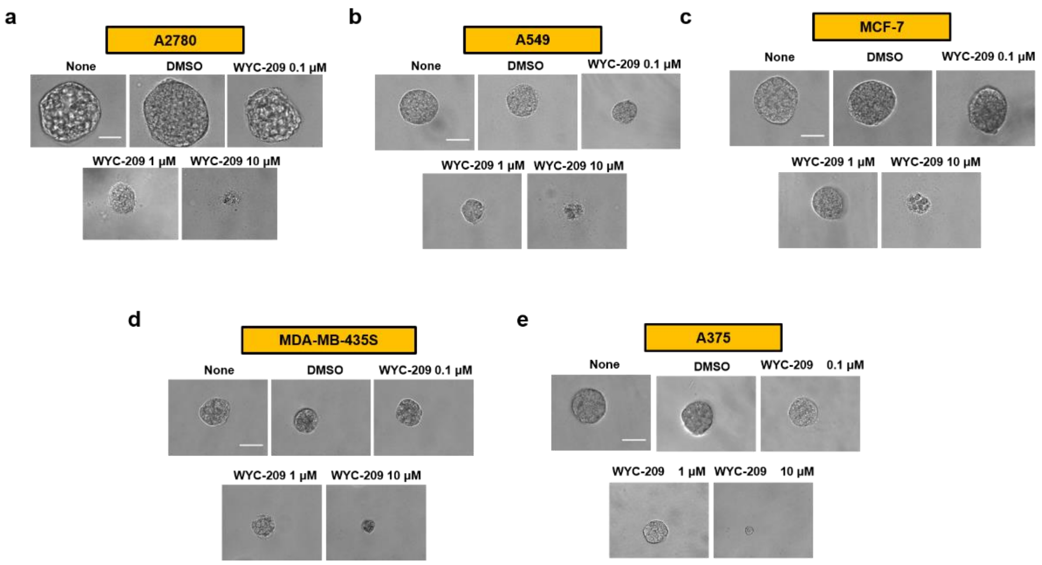

Supplementary Figure 16. WYC-209 inhibits colony growth of TRCs of 5 human cancer cell lines. Human ovarian cancer A2780 (**a**), human lung cancer A549 (**b**), human breast cancer MCF-7 (**c**) , human melanoma MDA-MB-435s (**d**) and human malignant melanoma A375 (**e**) were cultured in 90-Pa fibrin gels for 3 days and treated with 0.1, 1, or 10 μM WYC-209. **None**: medium only; **DMSO**: medium with 0.1% DMSO. Representative images of colony size on day 7 were shown. Note that different cell lines have different cell doubling times such that colony sizes are different. Scale bars, 50 μm.

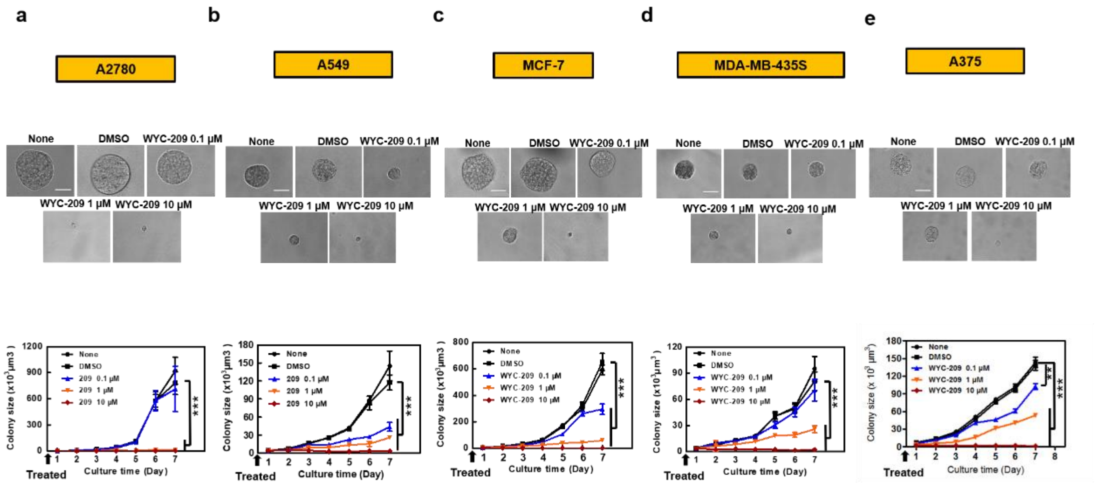

Supplementary Figure 17. WYC-209 blocks colony growth of TRCs of human ovarian, lung, breast and melanoma cells. Human ovarian cancer A2780 (**a**) , human lung cancer A549 (**b**), human breast cancer MCF-7 (**c**), human melanoma MDA-MB-435s (**d**) and human malignant melanoma A375 (**e**) were cultured in 90-Pa fibrin gels and treated with 0.1, 1, or 10 μM WYC-209 on day 0. **None**: medium only; **DMSO**: medium containing 0.1% DMSO. Mean ±s.e.m.; n=15 samples; 3 separate experiments; *** P<0.001 (Student's t-test). Bright field images were representative images of colonies in (**a**-**e**) on day 7 respectively. Scale bars, 50 μm.

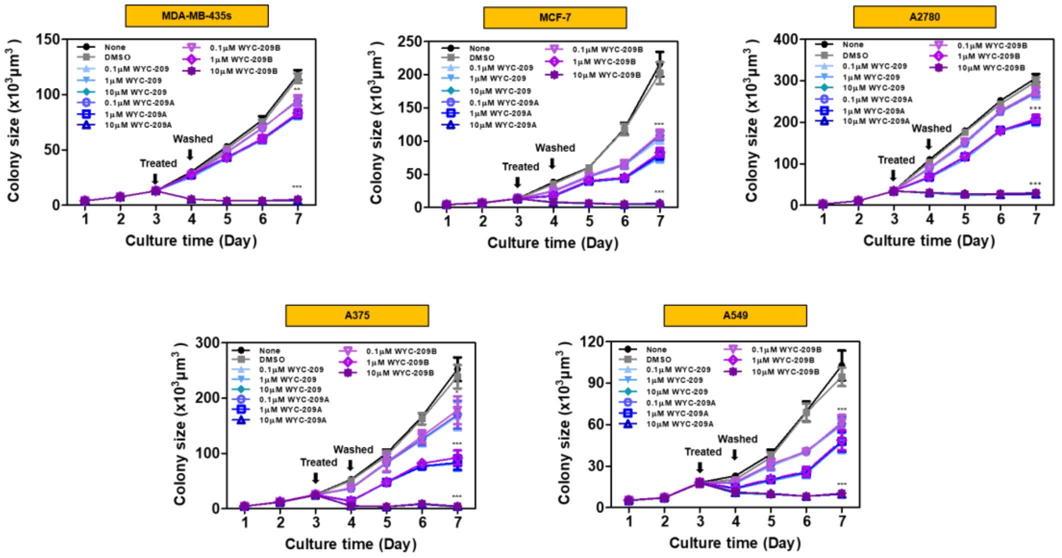

Supplementary Figure 18. WYC-209 inhibits colony growth of human cancer cells after washout. human melanoma MDA-MB-435s, human breast cancer MCF-7, human ovarian cancer A2780, human malignant melanoma A375, and human lung cancer A549 were cultured in 90-Pa 3D fibrin gels for 3 days, treated with 0.1% DMSO; 0.1, 1, or 10 µM WYC-209, WYC-209A, or WYC-209B on day 3, and then washed out of the compound on day 4 and cultured till day 7 day. Mean ± s.e.m.; n= 15 samples; 3 separate experiments. * P<0.05; ** P<0.01; *** P<0.001 (Student's t-test).

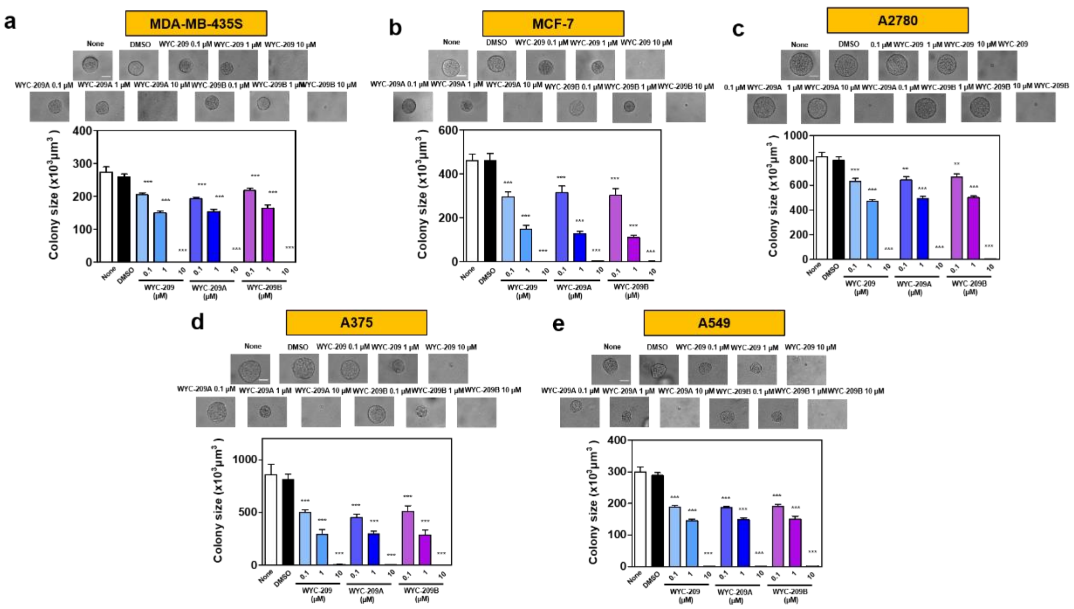

Supplementary Figure 19. WYC-209 abrogates colony growth of human TRCs after re-plating. (**a-e**): human melanoma MDA-MB-435s (**a**), human breast cancer MCF-7 (**b**), human ovarian cancer A2780 (**c**), human malignant melanoma A375 (**d**), and human lung cancer A549 (**e**) cultured in 90-Pa 3D fibrin gels and treated with various compounds on day 3, washed out on day 4, and isolated on day7, and then re-plated into 90-Pa 3D fibrin gels as single individual cells for 7 days without treatment. Top of each subfigure: Representative images of colonies on day 7 (all colonies started as a single cell on day 0); Scale bar, 50 μm. Mean ± s.e.m.; n= 15 samples; 3 separate experiments. ** *P*<0.01; *** *P*<0.001 (Student's t-test). Bright field images were representative images of colonies in (**a-e**) on day 7 respectively after re-plating. Scale bars, 50 μm.

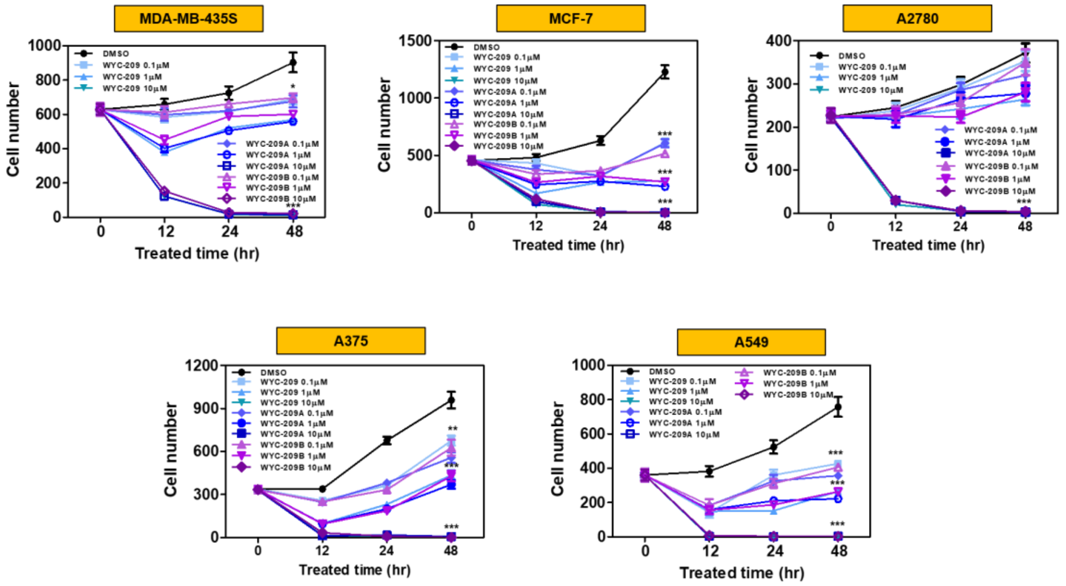

Supplementary Figure 20. WYC-209 detaches adherent human cancer cells on rigid plastic. (**a-e**): human melanoma MDA-MB-435s (**a**), human breast cancer MCF-7 (**b**), human ovarian cancer A2780 (**c**), human malignant melanoma A375 (**d**), and human lung cancer A549 (**e**) were cultured on rigid plastics and treated with 0.1, 1, or 10 μM WYC-209, WYC-209A, or WYC-209B and cell numbers were measured for 48 hours. Cell nuclei were stained with DAPI. **None**: medium only; **DMSO**: medium containing 0.1% DMSO. Cell numbers per view field were quantified. Mean ±s.e.m.; n= 5 samples; 3 separate experiments; $^{*}P<0.05$; $^{**}P<0.01$; $^{***}P<0.001$ (Student's t-test).

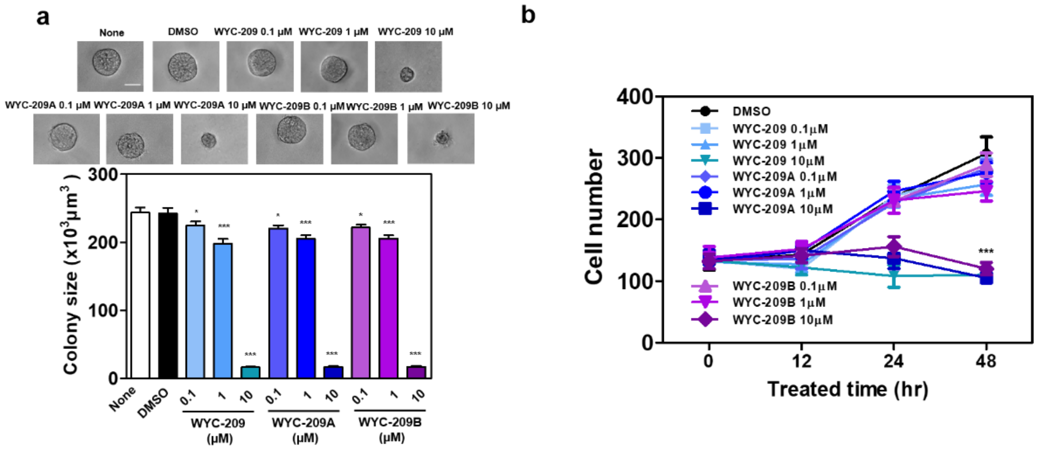

Supplementary Figure 21. WYC-209 has less inhibitory effects on non-cancerous human epidermal cell line HaCaT. (**a**) Non-cancerous human epidermal HaCaT cells were seeded into 3D soft fibrin gel and treated with 0.1, 1, or 10 μM of WYC-209, WYC-209A, or WYC-209B on day 0, and colony sizes were quantified on day 7. Bright field images were representative images of colonies on day 7. Scale bars, 50 μm. (**b**) Quantitation of cell numbers per view field of HaCaT cells plated on 2D rigid plastic. Cell nucleus were stained with DAPI. **None**: medium only; **DMSO**: medium containing 0.1% DMSO. Mean ± s.e.m.; n= 5 samples; 3 separate experiments; * *P*<0.05, *** *P*<0.001 (Student's t-test). Note that even at 10 μM concentration no reduction in cell numbers is observed on 2D and there is still some colony growth in 3D in comparison with Supplementary Figs. 17 and 20, suggesting that the compounds have less inhibitory effects on these non-cancerous human epidermal cells.

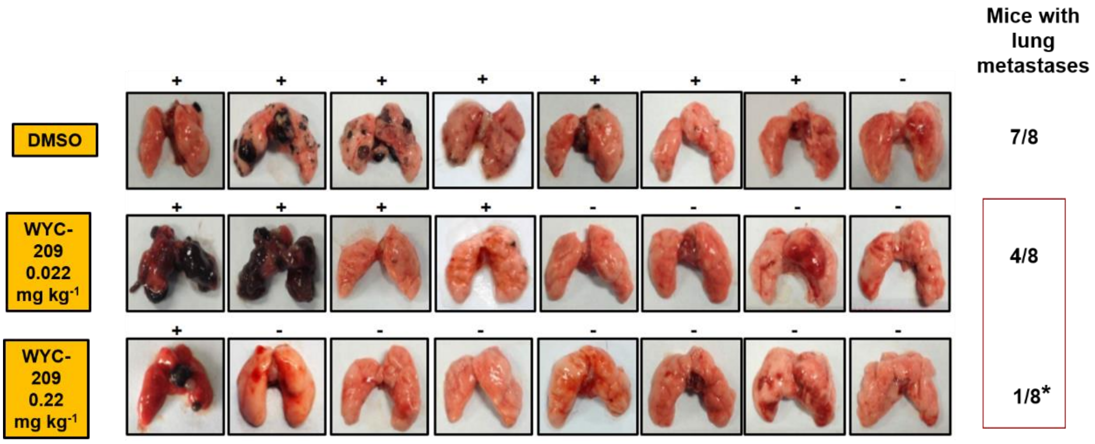

Supplementary Figure 22. Images of lungs in Fig. 5a up to day 30. “+”: lungs with tumor metastasis; “-”: lungs without tumor metastasis. Fisher’s exact test was used for statistical analysis,* P<0.05, compared with DMSO group.

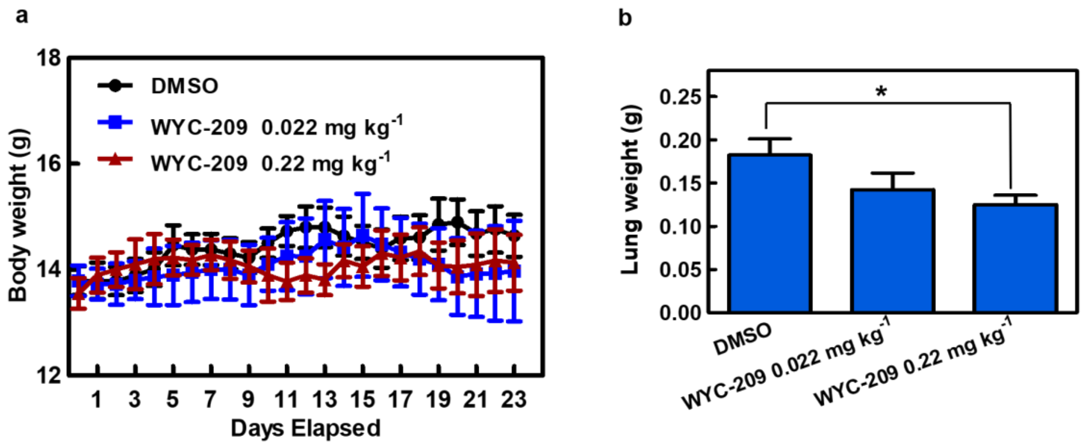

Supplementary Figure 23. WYC-209 has no effects on mouse weight. (**a**) Body weights of mice in Fig. 5 were recorded every day. There are no significant differences among groups. No abnormalities in mouse organs such as stomach, small intestines, brain, or liver were observed after WYC-209 treatment (not shown), suggesting little toxicity. (**b**) Quantitation of lung weights of mice at the time of scarification. Mean ± s.e.m.; n=8 mice in each group; * $P<0.05$ (Student's t-test). Low lung weights after treatment with 0.22 mg kg^{-1} WYC-209 are consistent with the data that lung metastases were found from only 1 mouse (see Fig. 5b).

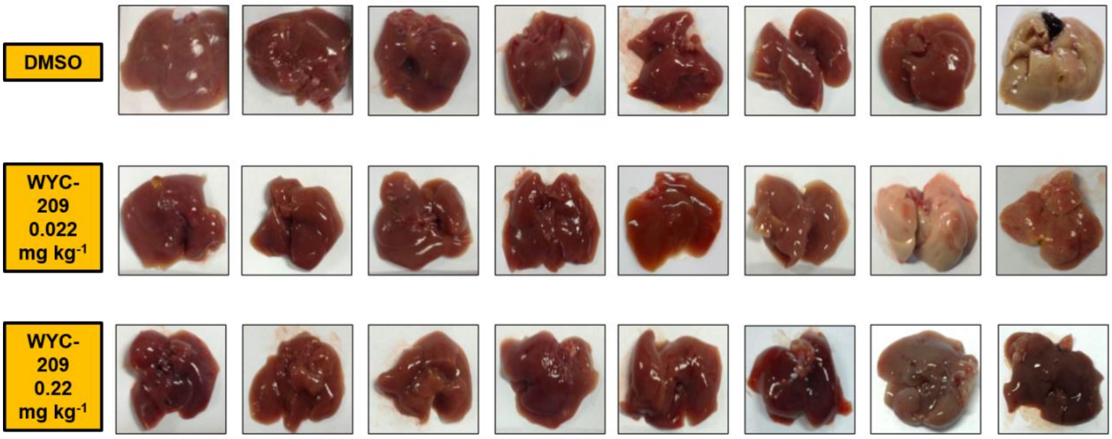

Supplementary Figure 24. WYC-209 has no toxic effects on mouse livers. Mice were treated using the same protocol as in Fig. 5a. Mice were dissected on day 30 or on the day of death. Note that there appears to be one liver (the panel on the rightest) in the DMSO group that has metastases and the mouse was already dead for several hours before dissection such that the color of the liver was different from the others.

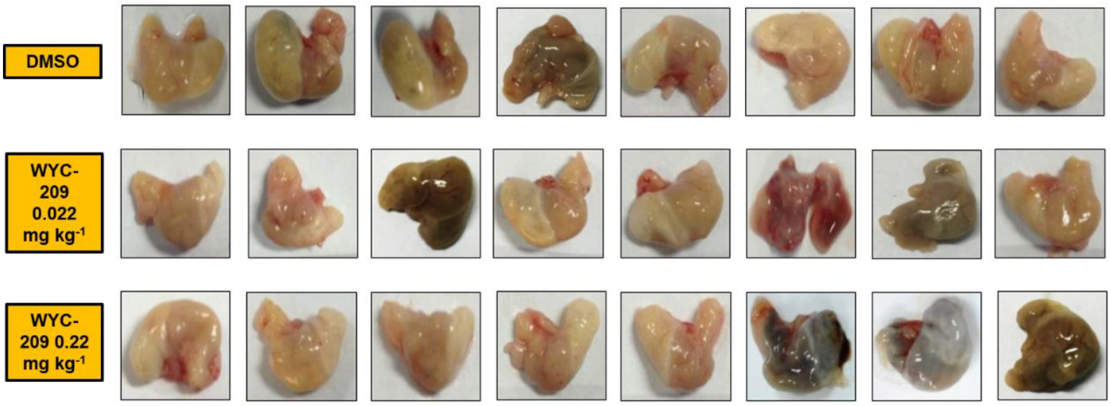

Supplementary Figure 25. WYC-209 has no toxic effects on mouse stomach. Mice were treated using the same protocol as in Fig. 5a. Mice were dissected on day 30 or on the day of death.

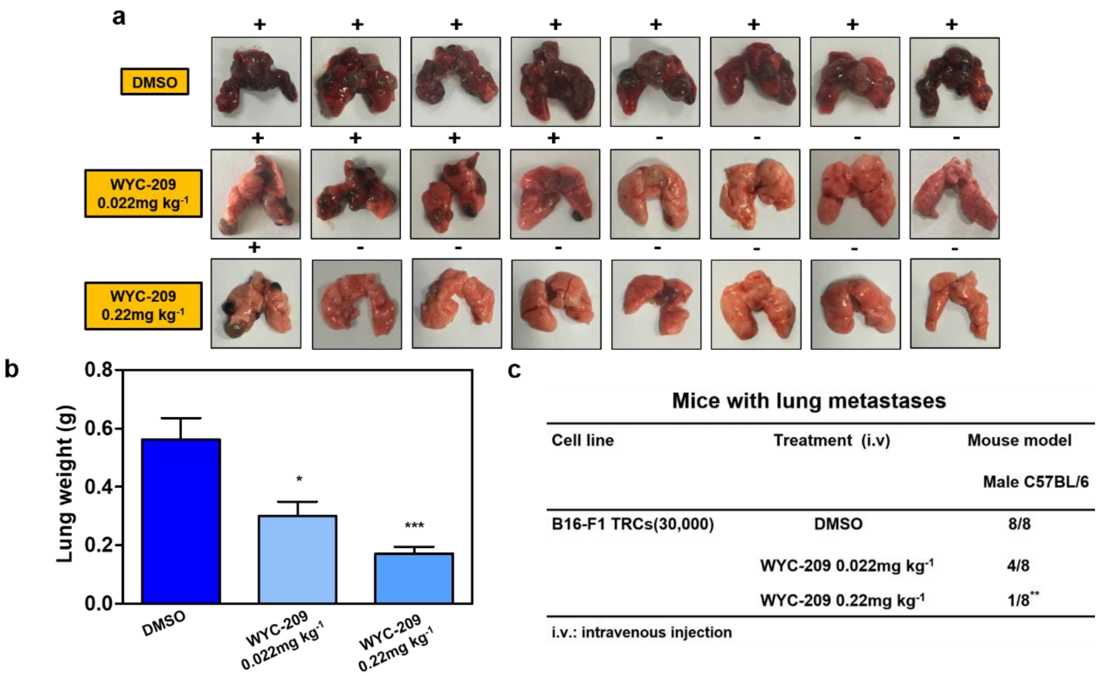

Supplementary Figure 26. WYC-209 inhibits tumor metastasis in male mice. Control B16-F1 cells were cultured in 90-Pa fibrin gels for 5 days to form TRCs. TRCs were isolated from gels and injected intravenously via tail veins into wild-type immune-competent syngeneic C57BL/6 male mice at 30,000 cells per mouse. **DMSO group**: 8 mice were injected with TRCs for 5 days and then were treated with DMSO (0.1%, vehicle) every two days via intravenous injection. **WYC-209 0.022 mg kg^{-1}**: 8 mice were injected with TRCs for 5 days then treated with WYC-209A 0.022 mg kg^{-1} every two days by intravenous injection. **WYC-209 0.22 mg kg^{-1}**: 8 mice were injected with TRCs for 5 days and then were treated with WYC-209A 0.22 mg kg^{-1} every two days by intravenous injection. Mice were dissected on day 30 or on the day of death to examine signs of lung metastases. (**a**) Photos of lungs in each group. (**b**) Quantitation of lung weights of mice at the time of scarification. Mean ± s.e.m.; n=8 mice per group; $^{*}P<0.05$; $^{***}P<0.001$ (Student's t-test). (**c**) Lung metastasis frequency of B16-F1 TRCs after treated with DMSO, WYC-209 0.022 mgkg^{-1}, WYC-209 0.22 mgkg^{-1}. $^{**}P<0.01$ with Fisher’s exact test.

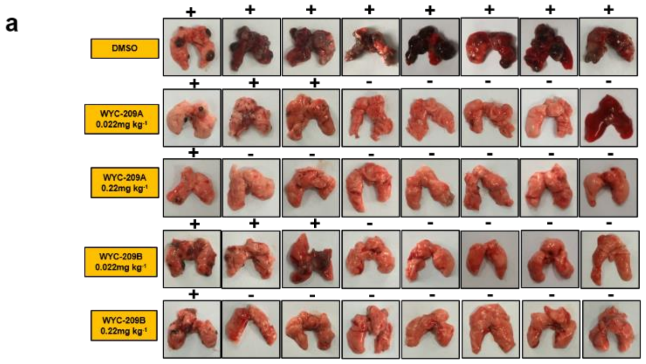

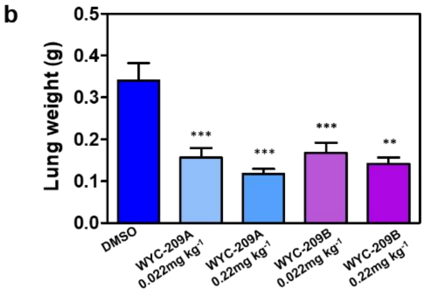

c

Mice with lung metastases

Cell line	Treatment (i.v)	Mouse model
		C57BL/6
B16-F1 TRCs(30,000)	DMSO	8/8
	WYC-209A 0.022 mg kg^{-1}	3/8*
	WYC-209A 0.22 mg kg^{-1}	1/8**
	WYC-209B 0.022 mg kg^{-1}	3/8*
	WYC-209B 0.22 mg kg^{-1}	1/8**

i.v.: intravenous injection

Supplementary Figure 27. WYC 209A and 209B inhibit tumor metastasis *in vivo.* Control B16-F1 cells were cultured in 90-Pa fibrin gels for 5 days to form TRCs. TRCs were isolated from gels and injected intravenously via tail veins into wild-type immune-competent syngeneic C57BL/6 mice at 30,000 cells per mouse. **DMSO group**: 8 mice were injected with TRCs for 5 days and then were treated with DMSO (0.1%, vehicle) every two days via intravenous injection. **WYC-209A** or **WYC-209B** at **0.022 mg kg^{-1}** or **0.22 mg kg^{-1}** were delivered every two days by intravenous injection after TRCs were injected intravenously for 5 days. Mice were dissected on day 30 or on the day of death to examine signs of lung metastases. (**b**) Quantitation of lung weights of mice at the time of scarification. Mean ± s.e.m.; n=8 mice per group; $^{**}P$<0.01; $^{***}P$<0.001 (Student's t-test). (**c**) Lung metastasis frequency of B16-F1 TRCs after various treatments. $^{*}P$<0.05, $^{**}P$<0.01 with Fisher's exact test.

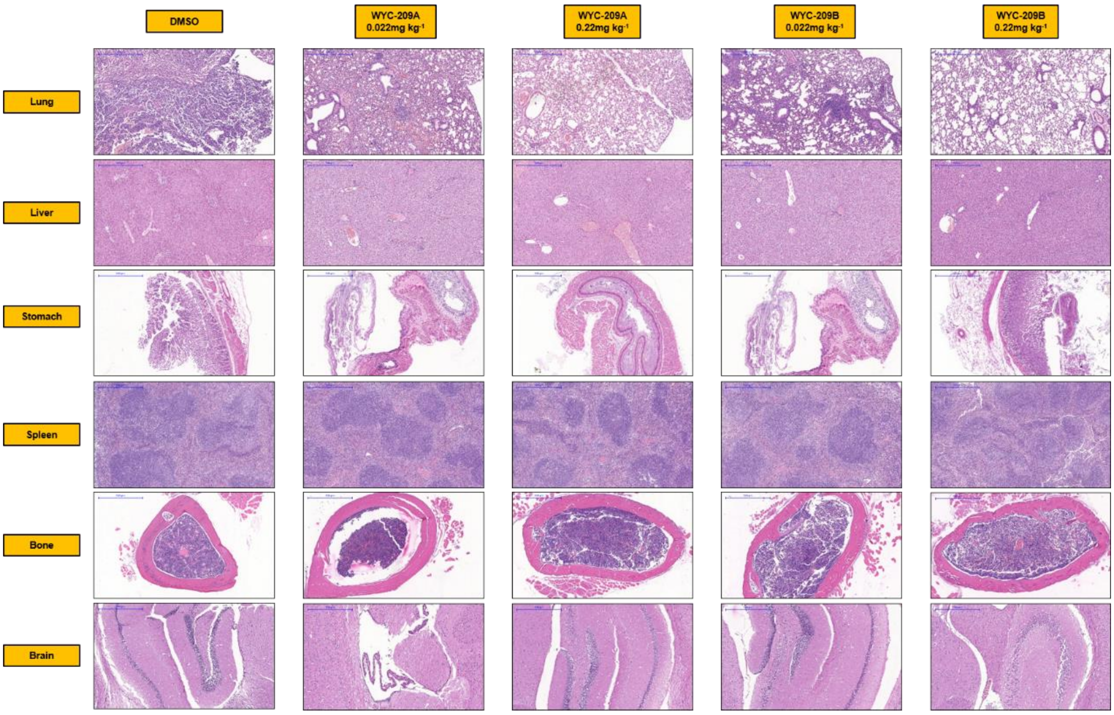

Supplementary Figure 28. H&E staining of mouse tissues. Representative hematoxylin and eosin (H&E)-stained sections of lung, liver, stomach, spleen, bone and brain from C57BL/6 mice intravenously injected with 30,000 TRCs per mouse. The mice were dissected on day 30 or on the day of death to examine signs of metastases. **DMSO**, **WYC-209A** or **WYC-209B** at **0.022 mg kg^{-1}** or **0.22 mg kg^{-1}**: 8 mice were injected with TRCs for 5 days and then were treated with 0.1% DMSO or the compound every two days via intravenous injection. All images were taken at 5X magnification; all scale bars, 500 μm. Three random sections per organ were performed. Mice in the DMSO group and the 0.022 mg kg^{-1} group of WYC-209A or WYC-209B exhibited metastases in the lungs but not in other organs. However, the size and the number of lung metastases in the WYC-209A or WYC-209B treated group were much less than those in the DMSO group. Mice in the 0.22 mg kg^{-1} group of WYC-209A or WYC-209B exhibited no metastases in the lungs and in other organs.

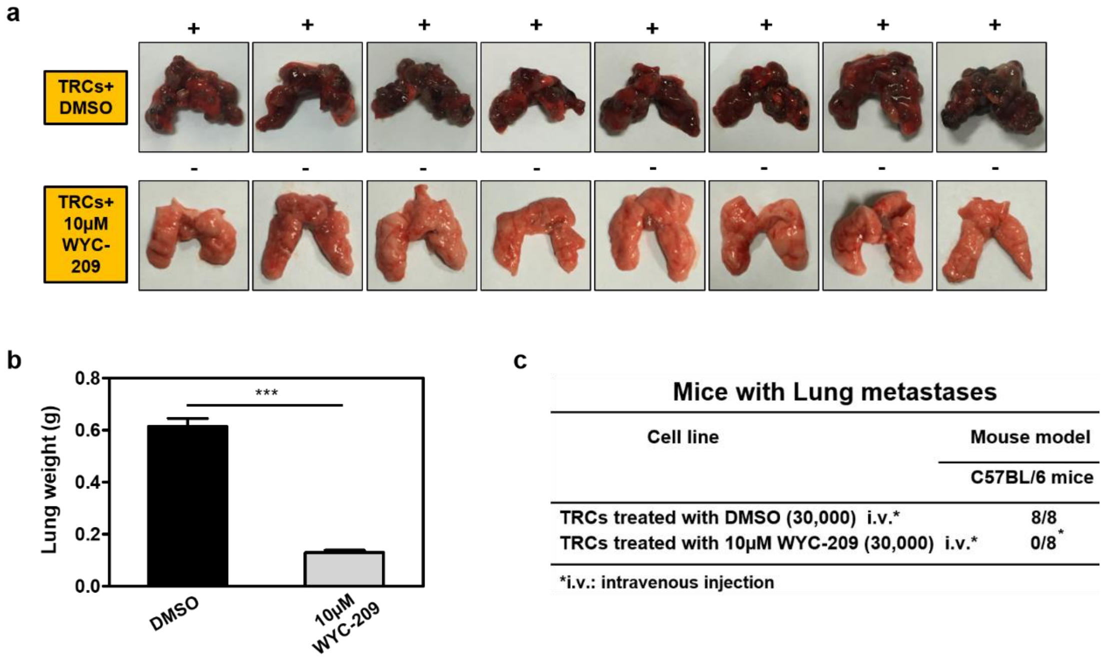

Supplementary Figure 29. WYC-209 treated TRCs in culture do not form tumor metastases *in vivo.* B16-F1 cells were cultured in 90-Pa fibrin gels and then treated with DMSO (0.1% DMSO-containing medium) or 10 μM WYC-209 on day 3. We then isolated the TRCs on day5 and injected intravenously via tail veins into wild-type immune-competent syngeneic C57BL/6 mice at 30,000 cells per mouse, 8 mice per group. (**a**) Images of the lungs on day 30. (**b**) Quantitation of lung weights of mice at the time of scarification. Mean ± s.e.m.; n=8 mice in each group; *** P<0.001 (Student's t-test). (**c**) Lung metastasis frequency of B16-F1 TRCs after treated with DMSO or 10 μM WYC-209. $^{*}P$<0.05 with Fisher's exact test.

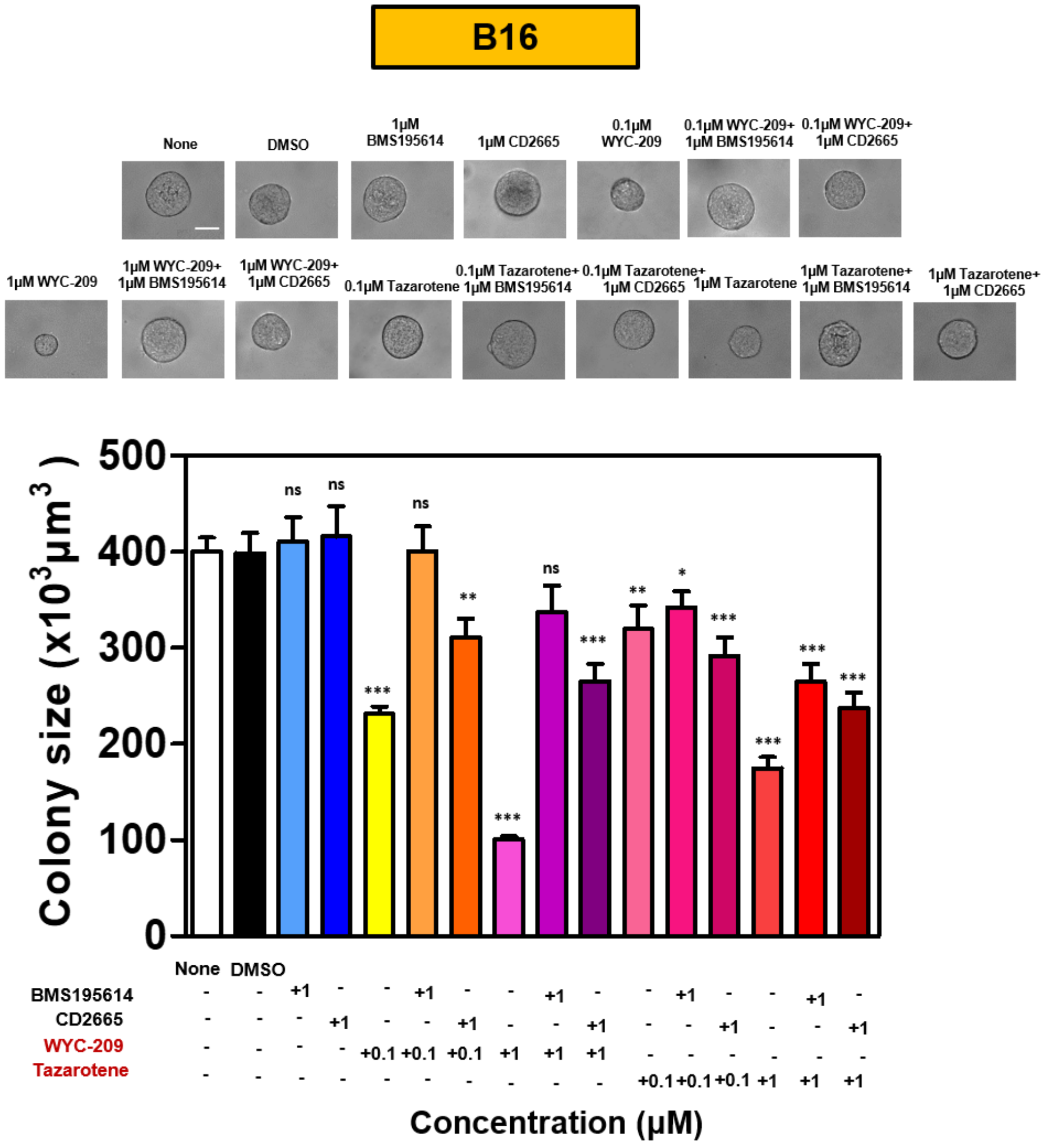

Supplementary Figure 30. Tazarotene inhibits TRC growth less than WYC-209. Control B16-F1 cells were cultured in 90-Pa fibrin gels treated with RA receptor antagonists for 3 h, then treated with 0.1 or 1 μM WYC-209, 0.1 or 1 μM Tazarotene and cultured for 5 days. Summarized colony sizes after drug treatments on day 5 were compared with DMSO groups. **None**: medium without drugs. **DMSO**: medium with 0.1% DMSO. **BMS 195614**: Neutral retinoic acid receptor (RAR) α-selective antagonist. **CD 2665**: selective RARβ/γ antagonist. Summarized colony sizes after drug treatments on day 5 were compared with DMSO groups. **None**: medium without drugs. Mean ± s.e.m.; n=15 samples; 3 separate experiments; ns, not significantly different; * P<0.05; ** P<0.01; *** P<0.001 (Student's t-test). Bright field images were representative images of colonies on day 5. Scale bar, 50 μm.

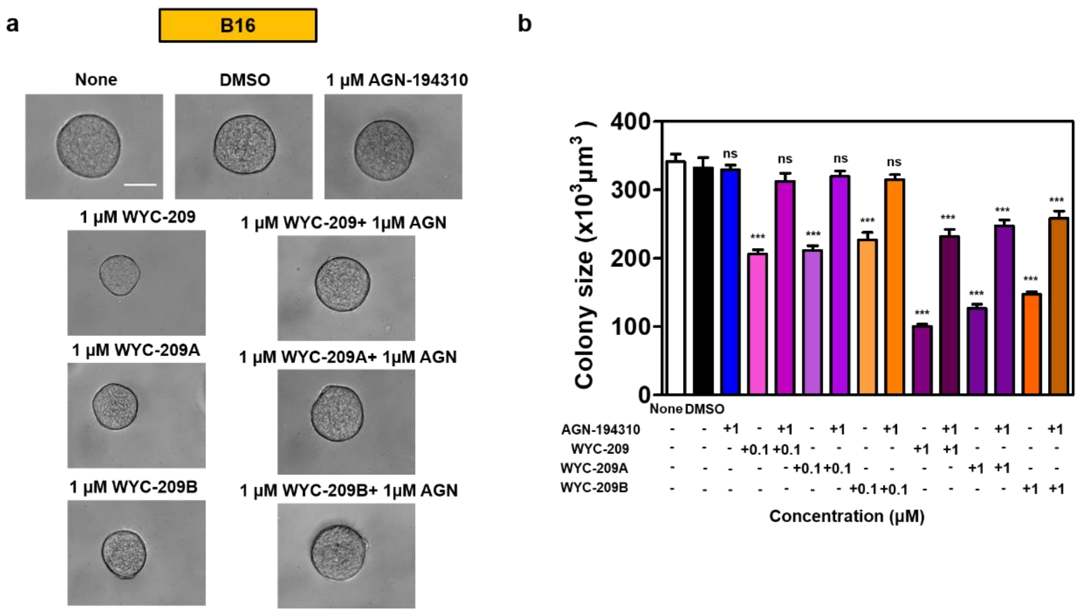

Supplementary Figure 31. Pan-antagonist of RAR rescues TRC growth from inhibitory effects of WYC-209 and WYC-209A/B. Control B16-F1 cells were cultured in 90-Pa fibrin gels treated with RA receptor pan-antagonists for 3 h, then treated with 0.1 or 1 μM WYC-209, and cultured for 5 days. Summarized colony sizes after drug treatments on day 5 were compared with DMSO groups. **None**: medium without drugs. **DMSO**: medium with 0.1% DMSO. **AGN-194310**: pan-antagonist of RAR. (**a**) Representative images of TRCs in different conditions at day 5. Bright field images were representative images of colonies on day 5. Scale bars, 50 μm. (**b**) Quantification of colony size of TRCs in different conditions at day 5. Mean ± s.e.m.; n=15 samples; 3 separate experiments; ns, not significantly different; *** P<0.001 (Student's t-test).

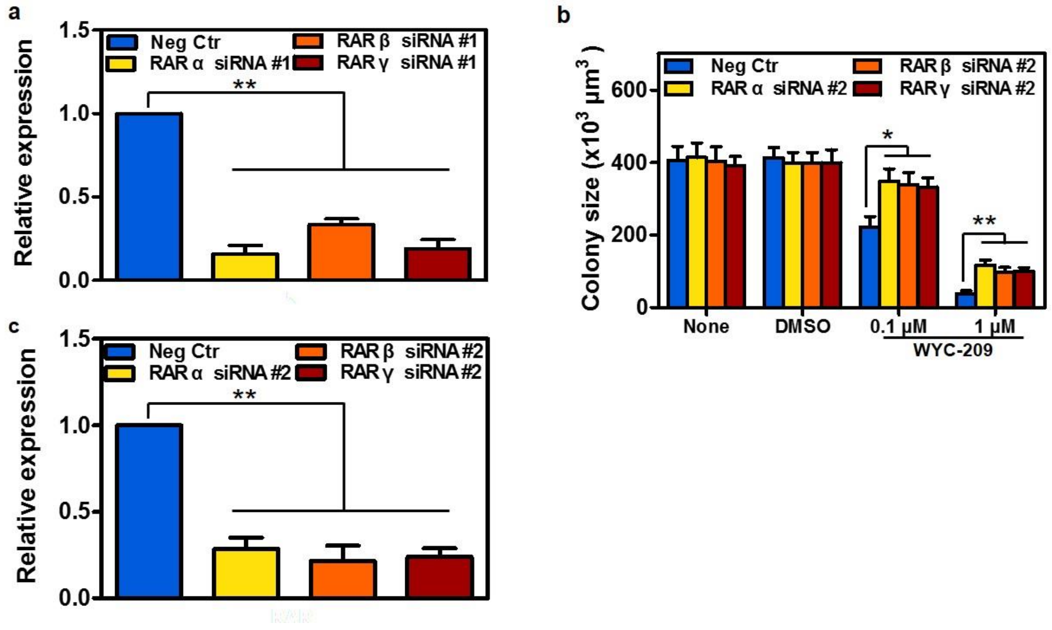

Supplementary Figure 32. Knocking down RAR rescues TRC growth from inhibitory effects of WYC-209. (**a**) Knocking down efficiency of siRNA #1. Control B16-F1 cells were transfected with negative control, RARα #1, RARβ #1, or RARγ #1 siRNAs respectively, and RT-qPCR data were performed to confirm the knockdown efficiency. (**b**) Control B16-F1 cells were transfected with negative control (scrambled siRNA), RARα, RARβ, or RARγ siRNA #2, respectively, and were then re-plated in soft fibrin gels, and were treated with 0.1 μM or 1 μM WYC-209. Summarized colony sizes after drug treatments on day 5 were compared with Negative control groups. **None**: medium without drugs. **DMSO**: medium with 0.1% DMSO. **Neg Ctr**: negative control. (**c**) Knocking down efficiency of siRNA #2s. Control B16-F1 cells were transfected with negative control, RARα, RARβ, or RARγ siRNA #2 respectively, and RT-qPCR data were performed to confirm the knockdown efficiency. Mean ± s.e.m.; 3 separate experiments; * P<0.05; ** P<0.01 (Student's t-test).

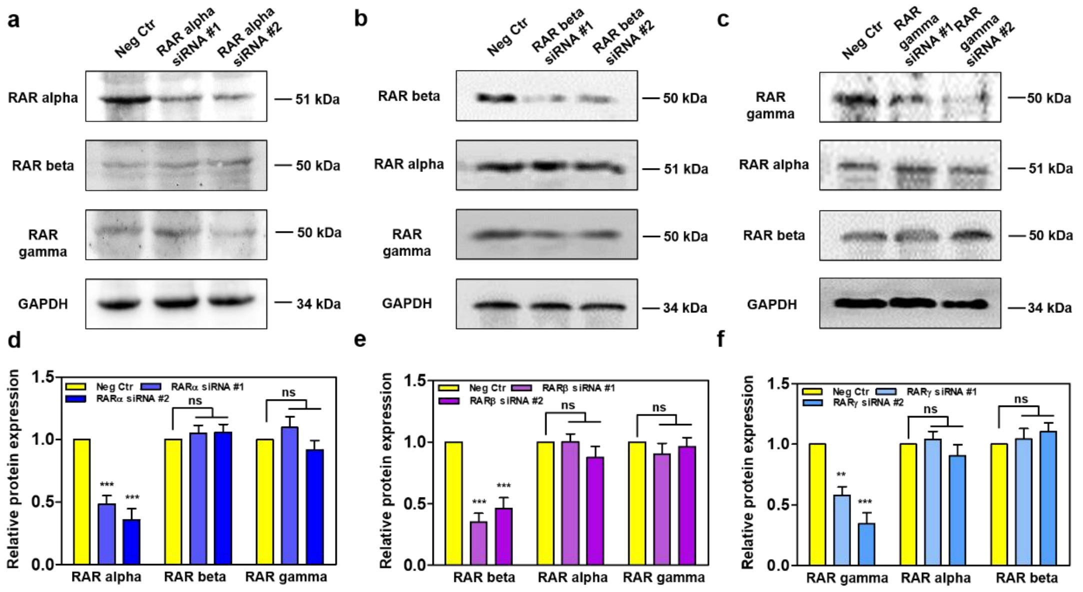

Supplementary Figure 33. Silencing efficiency and specificity of siRNAs to RARs. B16-F1 cells were transfected with RARα siRNA#1 and siRNA#2 (**a**), RARβ siRNA#1 and siRNA#2 (**b**), RARγ siRNA#1 and siRNA#2 (**c**). Protein levels of RAR alpha, beta and gamma were measured. (**a-c**) Representative images of Western blotting of various proteins expressions after siRNAs knockdown. **Neg Ctr:** treated with scrambled siRNA. Two different siRNAs (#1, #2) were used. (**d-f**) Quantification of silencing specificity of one subtype on protein levels of other subtypes. Mean ± s.e.m.; 3 separate experiments; ns, not significantly different; ** *P*<0.01; *** *P*<0.001 (Student's t-test).

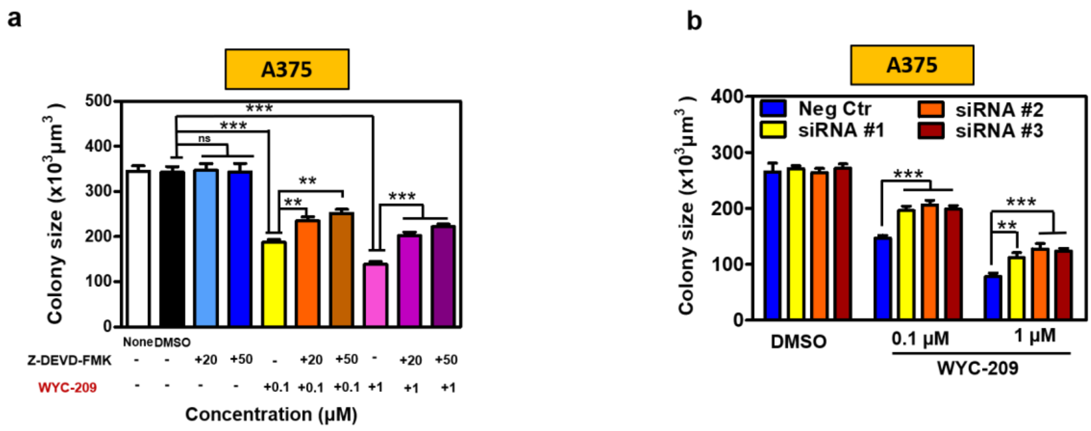

Supplementary Figure 34. WYC-209 inhibits A375-TRCs growth via the caspase 3 pathway. (a) Control A375 cells were cultured in 90-Pa fibrin gels treated with caspase 3 inhibitor Z-DEVD-FMK (a,b) for 3 hours, then treated with 0.1 or 1 µM WYC-209, and cultured for 7 days. Summarized colony sizes after drug treatments on day 7 were compared with DMSO groups. None: medium without drugs. DMSO: medium with 0.1% DMSO. Z-DEVD-FMK: caspase-3 inhibitor. (**b**) Control A375 cells were transfected with negative control (scrambled siRNA), caspase 3 siRNA #1, #2, or #3, respectively, re-plated in soft fibrin gels, and then were treated with 0.1 µM or 1 µM WYC-209. Summarized colony sizes after drug treatments on day 7 were compared with Negative control groups. **DMSO**: medium with 0.1% DMSO. **Neg Ctr**: negative control. Mean ± s.e.m.; n=15 samples; 3 separate experiments; ns: not significantly different; ** P<0.01; *** P<0.001 (Student's t-test with Bonferroni correction).

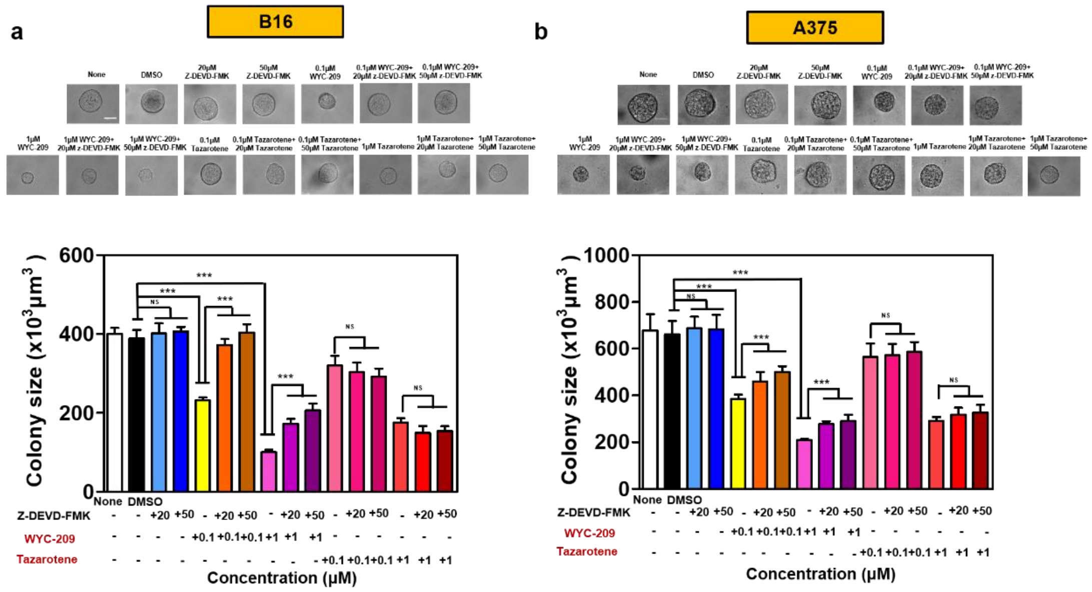

Supplementary Figure 35. Tazarotene inhibits TRC growth not via the caspase 3 pathway. (**a**) Control murine B16-F1 cells or (**b**) human A375 cells were cultured in 90-Pa fibrin gels, treated with caspase 3 inhibitor Z-DEVD-FMK for 3 hours, and then treated with 0.1 or 1 μM WYC-209, 0.1 or 1 μM Tazarotene and cultured for 5 days. Summarized colony sizes after drug treatments on day 5 (B16-F1) or day 7 (A375) were compared with DMSO groups. **None**: medium without drugs. **DMSO**: medium with 0.1% DMSO. **Z-DEVD-FMK**: caspase 3 inhibitor. Mean ± s.e.m.; n=15 samples; 3 separate experiments; ns, not significantly different; *** P<0.001 (Student's t-test with Bonferroni correction). Bright field images were representative images of colonies in (**a**, **b**) on day 5. Scale bars, 50 μm.

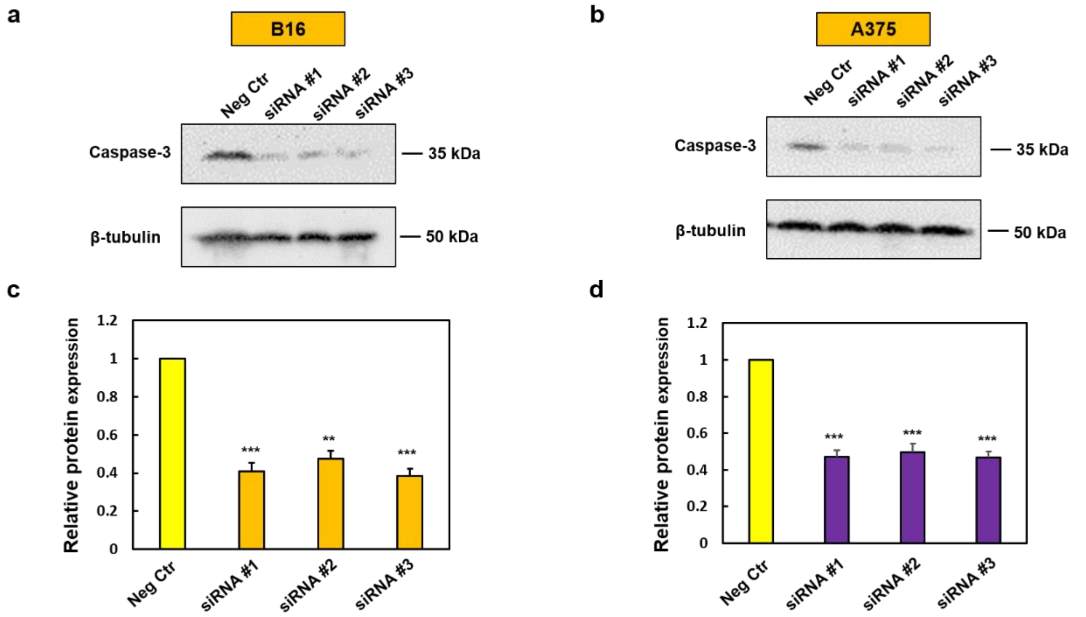

Supplementary Figure 36. Silencing efficiency of Caspase-3 siRNAs. Representative images of western blotting of Caspase-3 expression after siRNAs knockdown of B16-F1 (**a**) and A375 cells (**b**). **Neg Ctr**: treated with scrambled siRNA. Three different siRNAs (#1, #2 and #3) were used to exclude potential off-target effects. (**c, d**) Quantification of silencing efficiency on the protein level. All values are normalized by β-tubulin. Mean ± s.e.m.; 3 independent experiments; ** P<0.01; *** P<0.001 (Student's t-test).

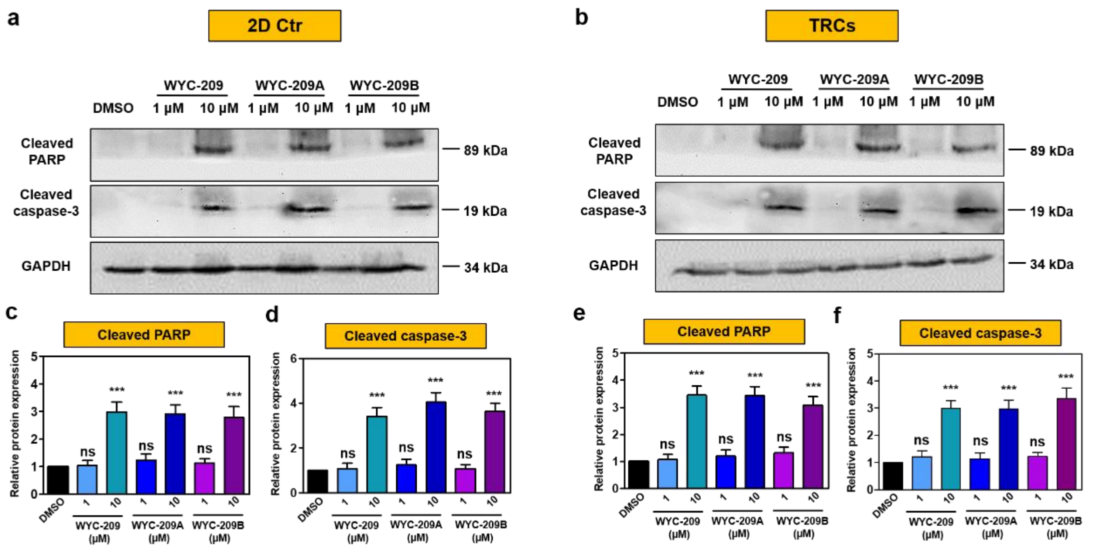

Supplementary Figure 37. WYC-209 induces cleaved caspase-3 and PARP expressions. (**a**) Representative images of cleaved PARP and cleaved caspase-3 protein expressions in control B16-F1 cells cultured on 2D rigid dishes and treated 0.1% DMSO, 1 or 10 μM WYC-209, WYC-209A or WYC-209B for 24 h. (**b**) Representative images of cleaved PARP and cleaved caspase-3 protein expression of control B16-F1 cells seeded in 3D soft fibrin gels for 4 days and treated 0.1% DMSO, 1 or 10 μM WYC-209, WYC-209A or WYC-209B for 24 hours. (**c, d**) Quantification of expression levels of cleaved PARP and cleaved caspase-3 in (**a**). (**e, f**) Quantification of expression levels of cleaved PARP and cleaved caspase-3 in (**b**). All values are normalized by GAPDH. Mean ± s.e.m.; 3 separate experiments; ns, not significantly different; *** *P*<0.001 (Student's t-test).

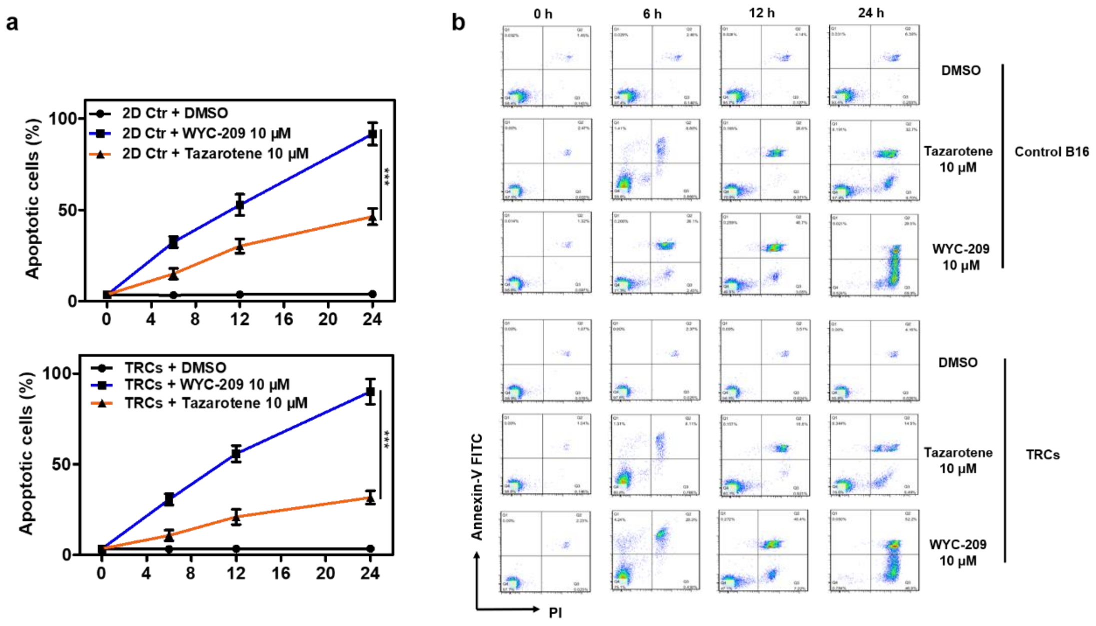

Supplementary Figure 38. WYC-209 induces tumor cell apoptosis. (**a**) Murine melanoma cells B16-F1, cultured on conventional rigid plates (**2D Ctr**) or seeded into 3D soft fibrin gels for 5 days (**TRCs**), were treated with 0.1% DMSO, 10 μM WYC-209, or 10 μM Tazarotene for indicated time periods. Then the cells were collected and stained with FITC-conjugated Annexin-V and PI for apoptotic detection by flow cytometry. Apoptotic cells (%) = 100% - (Annexin-V$^-$PI$^-$) %. Mean ± s.e.m.; 3 separate experiments; *** P<0.001 (Student's t-test). (**b**) Original images of the flow cytometry data in (**a**).

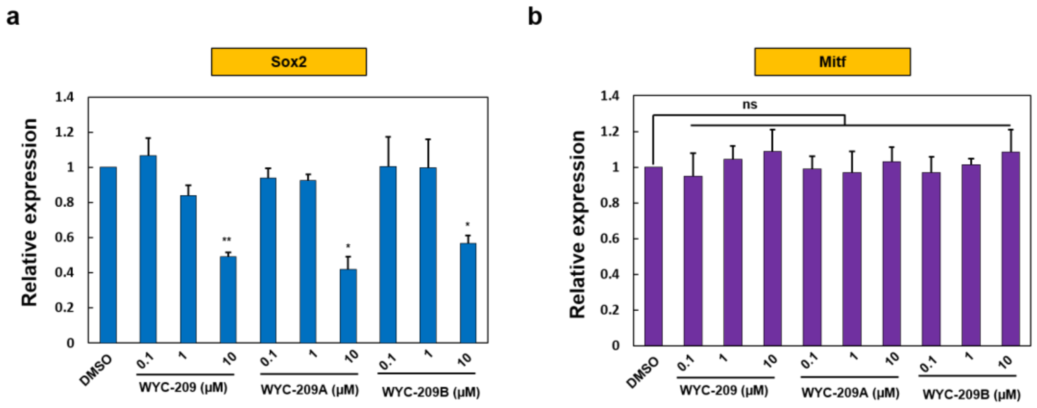

Supplementary Figure 39. WYC-209 decreases *Sox2* expression but has no effects on *Mitf* expression. Murine melanoma cells B16-F1 were seeded into 3D soft fibrin gels and then were treated with 0.1% DMSO, 0.1, 1 or 10 μM WYC-209, WYC-209A, or WYC-209B on day 4. mRNA of cells were extracted after 24 hours. *Sox2* (**a**) and *Mitf* (**b**) expressions were quantified by qPCR. Mean ±s.e.m.; 3 separate experiments; ns, not significantly different; * P<0.05; ** P<0.01 (Student's t-test).

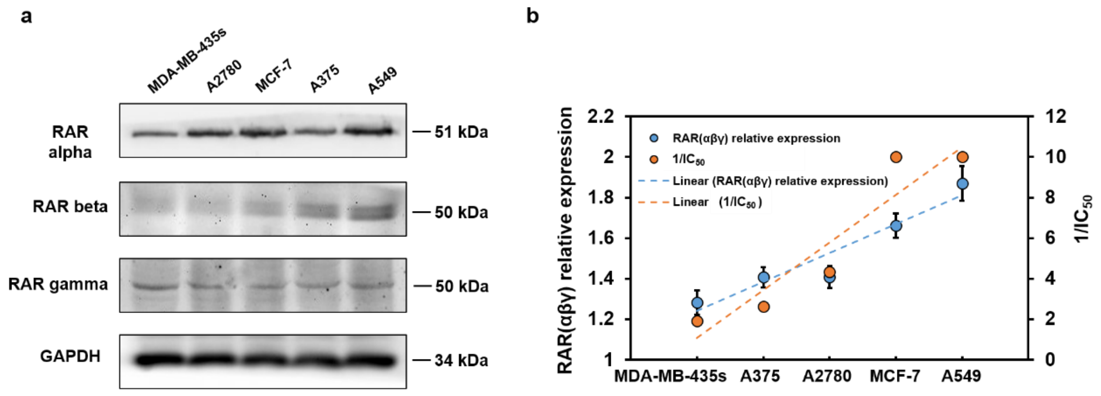

Supplementary Figure 40. RARs expression is correlated with sensitivity to WYC-209. (a) Representative images of western blotting of RAR αβγ expression in five human cancer cell lines. **(b)** RARs expression levels are positively correlated with sensitivity ($1/IC_{50}$) to WYC-209. RAR (αβγ) relative expression is the total gray levels of α, β, and γ relative to the gray value of GAPDH. Mean ± s.e.m.; n=3 independent experiments.

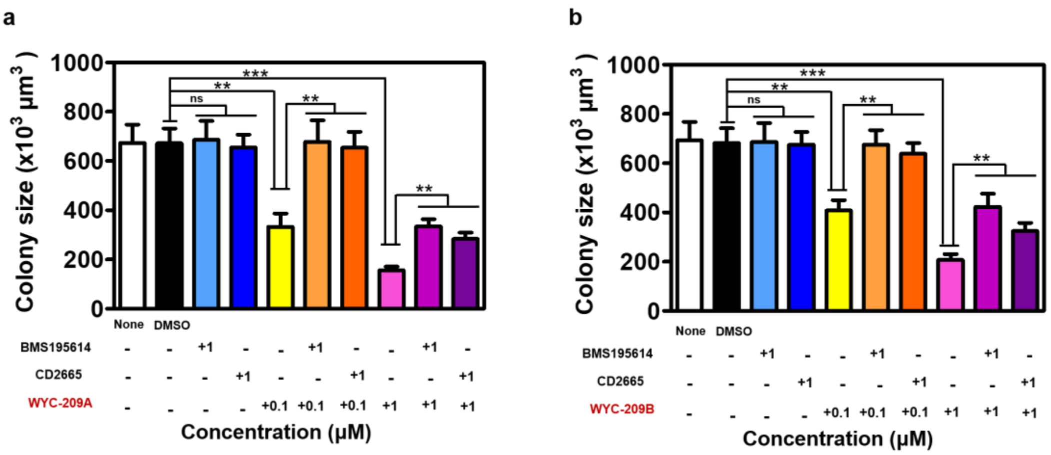

Supplementary Figure 41. WYC-209A and WYC-209B inhibit TRCs growth by binding to RA receptors. (**a, b**) Control B16-F1 cells were cultured in 90-Pa fibrin gels treated with RA receptor antagonists for 3 h, then treated with 0.1 or 1 µM WYC-209A (**a**) or WYC-209B (**b**), and cultured for 5 days. Summarized colony sizes after drug treatments on day 5 were compared with DMSO groups. **None**: medium without drugs. **DMSO**: medium with 0.1% DMSO. **BMS 195614**: retinoic acid receptor (RAR) α-selective antagonist. **CD2665**: selective RARβ/γ antagonist. Mean ± s.e.m.; n= 15 samples; 3 separate experiments; ns: not significantly different; ** P<0.01; *** P<0.001 (Student's t-test with Bonferroni correction).

Supplementary Table 1. Water solubility of WYC-209A and WYC-209B

Drugs	Test system	Solubility (μM)	
		Mean	Standard deviation
WYC-209A	PBS (pH 7.4)	**89.4**	0.06
WYC-209B	PBS (pH 7.4)	**88.1**	0.00
Propranolol (Control 1)	PBS (pH 7.4)	**>100 (119.3)**	0.01
Ketoconazole (Control 2)	PBS (pH 7.4)	**49.3**	0.17
Tamoxifen (Control 3)	PBS (pH 7.4)	**1.0**	0.09

Notes: Test concentration, 100 μM (1% of DMSO); Incubation condition, shaken (1000 rpm) for 1 h at room temperature; Sample size, Duplicates (n=2); Bioanalytical methods, liquid chromatography (LC)-mass spectrometry (MS).

Supplementary Table 2. K_d of WYC-209A and WYC-209B

Drug	K_d		
	**RAR*α***	**RAR*β***	**RAR*γ***
**WYC-209A acid**	5.3 nM	2.5 nM	0.53 nM
**WYC-209B acid**	1.3 nM	ND	ND
**ATRA**	0.91 nM	0.75 nM	1.96 nM

Notes: The binding affinity of WYC-209A acid, WYC-209B acid, or ATRA to human RAR*α*, RAR*β* and RAR*γ* was determined using the biophysical SPR protocol. ATRA, all-trans retinoic acid. The K_d values for the binding of the positive control ATRA are in the same order of magnitude as the published K_d values (0.20-0.36 nM) to mouse RARs[1]. ND: not determined. WYC-209A acid (99% e.e.) and WYC-209B acid (99% e.e.) were hydrolyzed from WYC-209A and WYC-209B and purified with chiral High Performance Liquid Chromatography.

Supplementary Table 3. IC_{50} of WYC-209 on human cancer cell lines.

Drug	Growth inhibition at 10μM	IC_{50}	Human Cell lines
WYC-209	100%	0.23 μM	A2780
WYC-209	100%	0.10 μM	A549
WYC-209	100%	0.10 μM	MCF-7
WYC-209	100%	0.52 μM	MDA-MB-435s
WYC-209	100%	0.38 μM	A375

Supplementary Table 4. hERG inhibition assay for WYC-209A and WYC-209B

Drugs	hERG IC_{50} (μM)
WYC-209A	> 30
WYC-209B	> 30
Cisapride (Control)	0.015

Supplementary Table 5. CYP inhibition assay for WYC-209A and WYC-209B

Drugs	CYP IC_{50} (μM)					
	1A2	2C9	2C19	2D6	3A4	3A4
WYC-209A	>10	>10	>10	>10	>10	>10
WYC-209B	>10	>10	>10	>10	>10	>10
Control	0.004	0.735	4.990	0.059	0.019	0.028

Notes: The substrate drugs used for CYP 1A2, 2C9, 2C19, 2D6, and 3A4 were phenacetin (30 μM), diclofenac (10 μM), S-mephenytoin (35 μM), bufuralol (10 μM) and testosterone (80 μM) respectively, while the control inhibitors for CYP 1A2, 2C9, 2C19, 2D6, and 3A4 were α-naphthoflavone (0.3 μM), sulfaphenazole (10 μM), omeprazole (100 μM), quinidine (2.5 μM), and ketoconazole (2.5 μM) respectively.

Supplementary Reference

1. Allenby, G. *et al.* Retinoic acid receptors and retinoid X receptors: interactions with endogenous retinoic acids. *Proc Natl Acad Sci U S A.* **90**, 30-34 (1993).
